# Microenvironmental IL13 Rα2 Expression and Enable Extracellular Vesicle-Mediated Antigen Transfer in Glioblastoma

**DOI:** 10.3390/cancers18213513

**Published:** 2026-10-31

**Authors:** Yujun Wang, Ryan Urak, Clara Chen, Martha Salas, Christine Brown, Lisa Anne Feldman

**Affiliations:** 1 Division of Neurosurgery, Department of Surgery, City of Hope National Medical Center and Beckman Research Institute, Duarte, CA 91010, USA; ywang@coh.org (Y.W.); clarachen08@gmail.com (C.C.); 2 Department of Hematology and Hematopoietic Cell Transplantation, T Cell Therapeutics Research Laboratories, City of Hope National Medical Center and Beckman Research Institute, Duarte, CA 91010, USA; rurak@coh.org (R.U.); cbrown@coh.org (C.B.); 3 Light Microscopy Core, City of Hope National Medical Center and Beckman Research Institute, Duarte, CA 91010, USA; masalas@coh.org

**Keywords:** glioblastoma, microenvironment, IL13Rα2 expression, extracellular vesicle, CAR T cells

## Abstract

**Background/Objectives:** Interleukin-13 receptor alpha 2 (IL13Rα2), expressed in more than 75% of glioblastomas (GBM), is a promising chimeric antigen receptor (CAR) T cell target. We investigated how microenvironmental stresses regulate IL13Rα2 expression and whether extracellular vesicle (EV)-mediated antigen transfer modulates CAR T cell killing. **Methods:** Patient-derived brain tumor and other GBM cell lines were exposed to hypoxia, desferrioxamine (DFO), or reduced fetal bovine serum (FBS). Western blotting assessed IL13Rα2 expression and recovery following reoxygenation. Fluorescence imaging evaluated EV interactions with recipient cells, and cytotoxicity assays assessed CAR T cell killing. **Results:** Hypoxia and DFO markedly suppressed IL13Rα2 expression. Exposure to 1% oxygen for 48 h reduced protein levels by 40–70%; expression recovered to near-baseline levels within 48 h of reoxygenation. Reduced FBS concentrations for 48 h also decreased IL13Rα2 expression compared with 10% FBS. Findings suggested potential contributions from ubiquitin-dependent degradation and transcriptional mechanisms. IL13Rα2-expressing EVs showed a slight increase under hypoxia. Fluorescence imaging confirmed that IL13Rα2-containing EVs attached to or entered recipient GBM cells within hours. EV-mediated antigen transfer increased CAR T cell killing by 3–5%. **Conclusions:** Hypoxia and nutrient availability dynamically regulate IL13Rα2 expression, while EVs can transfer the antigen between GBM cells and modestly enhance CAR T cell killing. These findings highlight reversible antigen modulation and EV-mediated antigen redistribution as mechanisms that could inform strategies to overcome antigen escape and improve immune targeting in GBM.

## Simple Summary

Interleukin-13 receptor alpha 2 (IL13Rα2) is regarded as a promising therapeutic target for glioblastoma (GBM) and has been extensively explored in chimeric antigen receptor T cell (CAR-T)-based immunotherapy. However, its heterogeneous expression within the GBM-specific microenvironment may influence therapeutic efficacy. The aim of our study was to assess how tumor microenvironmental stresses affect IL13Rα2 expression in glioma cell lines and whether IL13Rα2 transfer via extracellular vesicles can modulate CAR-T cell killing efficacy. We found that hypoxia and nutrient deprivation markedly suppressed IL13Rα2 expression in GBM cells, potentially through ubiquitin-dependent degradation pathways and transcriptional regulation. Expression could be restored when cells were returned to the original culture conditions. In addition, extracellular vesicles can deliver IL13Rα2, thereby enhancing the killing efficacy of CAR-T cells. Our findings deepen our understanding of the regulatory mechanisms governing IL13Rα2 expression and could inform strategies to overcome antigen escape in GBM therapy and enhance therapeutic efficacy in the clinic.

## 1. Introduction

Glioblastoma (GBM) is a highly aggressive primary brain tumor characterized by rapid growth, therapy resistance, and a diffuse infiltrative nature [1,2]. Despite maximal surgery and chemoradiotherapy, GBM invariably recurs, and patient prognosis remains poor, with an average survival of 12–15 months [3]. Targeted therapies are urgently needed to eliminate residual tumor cells while sparing normal brain tissue. One promising target in GBM is the interleukin-13 receptor alpha 2 (IL13Rα2), which promotes invasion and metastasis through the extracellular signal-regulated kinase/activator protein 1 pathway [4,5]. Immunohistochemistry studies have found IL13Rα2 protein in over half of IDH wild-type GBM patient samples (51–75% of cases) [6], particularly in the mesenchymal subtype [7]. In contrast, IL13Rα2 is not detected in healthy brain tissue [8]. Moreover, IL13Rα2 levels correlate with tumor grade and patient outcomes [7]; high IL13Rα2 expression is associated with increased tumor invasiveness and significantly shorter survival [9]. These observations establish IL13Rα2 as both a marker of aggressive disease and an appealing therapeutic target [10].

Several IL13Rα2-targeted therapies have been explored. Researchers initially targeted IL13Rα2 with fusion toxins, such as IL13-PE38QQR, which showed efficacy in preclinical models by exploiting IL13Rα2 internalization [11]. More recently, adoptive cellular immunotherapy against IL13Rα2 has demonstrated clinical potential. Chimeric antigen receptor (CAR) T cells specific for IL13Rα2 can selectively recognize and kill IL13Rα2-expressing GBM cells [7,12]. In a dramatic case report, sequential intracranial infusions of IL13Rα2-CAR T cells led to regression of all tumors in a patient with multifocal recurrent GBM, resulting in a near-complete response lasting 7.5 months [13]. Ongoing clinical trials have further tested IL13Rα2-CAR T therapy in larger cohorts. For example, a recent phase I trial treating 65 patients with locoregional IL13Rα2-CAR T cell delivery reported that the approach is feasible and safe, with preliminary signs of immune activity in the tumor microenvironment [12]. These studies underscore the therapeutic promise of IL13Rα2-directed CAR T cells and other IL13Rα2-targeted agents.

However, a significant challenge for IL13Rα2-targeted therapy is antigen heterogeneity and its modulation. Not all GBM cells uniformly express IL13Rα2, even within IL13Rα2-positive tumors, subpopulations can exhibit low or absent IL13Rα2 expression [14]. Therefore, IL13Rα2-targeted CAR T cells may fail to recognize and eliminate every tumor cell, allowing antigen-negative cells to survive and drive relapse [15]. Indeed, antigen escape has been observed clinically in trials of CAR T for other targets, in which tumor cells often recur with the loss or reduction of the target antigen [16,17,18]. In the context of IL13Rα2, the transient nature of some clinical responses suggests that the downregulation or loss of IL13Rα2 in residual cells may enable tumor regrowth by allowing escape from targeting. Even beyond genetic heterogeneity, the tumor microenvironment can dynamically influence antigen expression. Hypoxia [19,20] and other stresses in the GBM microenvironment, such as nutrient deprivation [21], are known to trigger broad changes in tumor cell gene expression and phenotype. For example, one study reported that acute hypoxic stress upregulated *IL13Rα2* mRNA levels in U-87 MG glioma cells [22], highlighting that IL13Rα2 expression is responsive to environmental conditions. Such microenvironment-induced modulation of IL13Rα2 could affect the efficacy of IL13Rα2-targeted therapies by transiently reducing antigen availability.

Intercellular communication within the tumor may also affect antigen presentation. GBM cells release abundant extracellular vesicles (EVs), including nano-sized exosomes, which carry proteins and RNAs that reflect the parent cell’s molecular state [15]. Hypoxia in particular can drive tumor cells to secrete exosomes enriched in hypoxia-regulated factors that promote angiogenesis and tumor aggressiveness [23,24]. We hypothesized that IL13Rα2 might be among the membrane proteins shed by tumor-derived EVs, with potentially significant biological and clinical implications. EV-mediated transfer of surface proteins between cells is a recognized phenomenon; in other cancers, tumor-derived exosomes can ferry antigens or immune modulators, such as exosomal PD-L1, contributing to T cell suppression [25,26]. In patients with schwannoma, neurofibroma [27], and GBM [28] tumors, IL13Rα2-containing exosomes have been detected in patient cerebrospinal fluid (CSF) and plasma, suggesting that tumor cells release IL13Rα2 into vesicles. If IL13Rα2 is packaged into EVs, this could, on the one hand, reduce antigen density on tumor cells (potentially hindering CAR T recognition), but, on the other hand, facilitate antigen enrichment in recipient cells through the transfer of IL13Rα2. Such a transfer might paradoxically broaden the reach of IL13Rα2-directed therapies, analogous to experimental strategies in which a “marker” antigen is introduced into tumors to make them visible to CAR T cells [29]. For instance, engineered oncolytic viruses expressing a truncated CD19 have been used to tag antigen-negative tumor cells with CD19, rendering them targetable by CD19 CAR T cells [29]. A natural form of this principle might occur if IL13Rα2-positive GBM cells secrete exosomes that fuse with neighboring cells, thereby distributing the IL13Rα2 protein.

In this study, we aimed to (1) define how microenvironmental stressors, including hypoxia and nutrient deprivation, regulate IL13Rα2 expression in GBM cells, including PBT cells; (2) quantify and characterize extracellular vesicles carrying IL13Rα2; and (3) evaluate the implications of these processes for IL13Rα2-targeted CAR T cell therapy. We report that hypoxic and nutrient-deprived conditions markedly downregulate IL13Rα2 in GBM cells, an effect that is reversible upon reoxygenation or restoration of full nutrients. Furthermore, we show that IL13Rα2 is present on GBM-derived vesicles, including exosomes, facilitating intercellular transfer. Functionally, the uptake of IL13Rα2-positive exosomes by patient-derived GBM tumor cells increases their susceptibility to killing by IL13Rα2-CAR T cells. These findings shed light on adaptive resistance mechanisms to IL13Rα2-directed therapy and reveal a potential endogenous mechanism for mitigating antigen escape via vesicle-mediated antigen dissemination.

## 2. Methods and Materials

### 2.1. Cell Lines and Culture Conditions

PBT030 and PBT138 are GBM neurosphere cell lines acquired from patient specimens at City of Hope under protocols approved by the Institutional Review Board, and they have been shown to preserve clinically relevant tumor characteristics [7,30]. The PBT030 tumor had first been expanded by two subcutaneous passages in mice prior to neurosphere culturing; and PBT138 had been engineered to express the enhanced green fluorescent protein (eGFP) and firefly luciferase reporter genes as previously described [31]. PBT138/IL13Rα2 is a PBT138 derivative that also stably overexpresses IL13Rα2. For neurosphere cultures, cells were maintained in DMEM/F12 (50/50) medium (Cat#: 10-092-CV, Thermo Fisher Scientific, Waltham, MA, USA), supplemented with 1× B27 supplement (Cat#: 17504-044, Thermo Fisher Scientific), heparin (Fresenius Kabi, 0.4 mL of 1000 USP units/mL in 500 mL of medium), and epidermal growth factor (EGF) and fibroblast growth factor (FGF) at final concentrations of 20 ng/mL. U-87 MG and U-251 MG cell lines were purchased from the American Type Culture Collection (ATCC, Manassas, VA, USA). Human primary astrocytes were derived from human cerebral cortex (Cell Applications Inc, San Diego, CA, USA). U-87 MG, U-251 MG, Astrocytes, and monolayer-cultured PBT030 cells (PBT030-2D) were grown in DMEM/F12 50/50 medium supplemented with 10% fetal bovine serum (FBS), 1% penicillin/streptomycin, and Plasmocin (Cat#: ant-mpp, InvitroGen, San Diego, CA, USA). Astrocytes were cultured in dishes coated with collagen I (Cat#: A10483-01, Thermo Fisher Scientific). IL13Rα2-specific CAR T cells [32] were cultured and resuspended in X Vivo15 medium (Cat#: 04-418, Lonza, Walkersville, MD, USA) containing 10% fetal calf serum (FCS), 50 U mL^−1^ rhIL-2 and 0.5 ng mL^−1^ rhIL-15. All cell lines used in this study were cultured in a humidified 5% CO_2_ incubator at 37 °C and were confirmed negative for Mycoplasma using the MycoAlertTM Mycoplasma Detection Kit (Cat#: LT07-318, Lonza) before initiating experiments.

### 2.2. Hypoxia and Oxygen Restoration Treatment

Tumor cells were seeded and cultured overnight to reach approximately 80% confluence before hypoxic exposure. Chemical hypoxia was induced with the hypoxia-mimetic desferrioxamine (DFO, Cat#: D9533, Millipore-Sigma, Burlington, MA, USA), an iron chelator that stabilizes HIF-1α. Cells were treated with DFO at 25, 50, 100, or 200 µM for 24, 48, or 72 h. Control cells were maintained under normoxic conditions (21% O_2_) without DFO.

Physical hypoxia was achieved using a modular hypoxia chamber (Cat#: 27310, Stemcell Technologies, Vancouver, BC, Canada). Cell culture dishes were placed inside the chamber and flushed with a gas mixture of either 1% O_2_, 5% CO_2_, and 94% N_2_ or 5% O_2_, 5% CO_2_, and 90% N_2_ for 15 min, per the manufacturer’s instructions. One hour later, the chamber was flushed again for 10 min. The chamber remained tightly sealed and was placed in a 37 °C cell culture incubator for 24 or 48 h. Normoxic cultures (21% O_2_) served as controls. For oxygen restoration experiments, hypoxia-treated cells were returned to normoxic conditions (standard culture conditions under approximately 21% O_2_) and cultured for an additional 24 or 48 h. Treated cells were then harvested to assess IL13Rα2 expression at the mRNA and protein levels.

### 2.3. FBS Deprivation and Restoration Treatment

Tumor cells were seeded and cultured overnight to reach approximately 80% confluence. For FBS deprivation, cells were washed with PBS three times and then cultured in fresh medium with varying concentrations of FBS (0%, 1%, 2%, 5%, 10% or 20%) for 24, 48, or 72 h. For FBS restoration experiments, serum-deprived cells were returned to complete growth medium containing 10% FBS and cultured for an additional 24 or 48 h. Cells were then harvested to assess IL13Rα2 expression at the mRNA and protein levels.

### 2.4. Western Blotting

Harvested cells were lysed on ice for 10 min using M-PerTM Mammalian Protein Extraction Reagent (Cat#: 78501, Thermo Fisher Scientific) supplemented with HaltTM Protease and Phosphatase Inhibitor Cocktail (Cat#: PI78440, Thermo Scientific). The protein lysates were cleared at 16,000× *g* for 10 min, resolved on NuPAGE 4–10% Bis-Tris gels (Cat#: NP0336BOX, InvitroGen), and transferred to PVDF membranes (Bio-Rad) using the Novex NuPAGE SDS-PAGE Gel System (InvitroGen). For immunoblotting, membranes were first blocked with 5% fat-free dry milk for 1 h at room temperature, then washed twice with Tris-Buffered Saline containing 0.5% Tween-20 (TBST). Membranes were subsequently incubated with primary antibodies overnight (Anti-IL13Rα2 from R&D Systems, Cat#: AF146, Minneapolis, MN, USA; Anti-HIF1A from Novus Biologicals, Cat#: NB100-105, Centennial, CO, USA; Anti-GAPDH (14C10) from Cell Signaling, Cat#: 2118S, Danvers, MA, USA), followed by three washes with TBST and a one-hour incubation with the corresponding secondary antibody. After TBST washing, membranes were incubated with SuperSignal West Pico Chemiluminescent substrate (Cat#: 34080, Thermo Scientific), and images were captured using the ChemiDoc imaging system (Bio-Rad) or developed on medical film using a SRX-101A processor (Konica Minolta, Tokyo, Japan). To compare gene expression, the density of Western blotting bands was measured using ImageJ software (ImageJ 1.54g), followed by calculation of the change compared with the corresponding controls, which were set to “1”.

### 2.5. Reverse Transcription and qRT–PCR Analysis

Total RNA was isolated from GBM cells using Trizol Reagent (Cat#: 15596026, Thermo Fisher Scientific) according to the manufacturer’s instructions. A total of 2.0 µg of total RNA underwent reverse transcription to determine mRNA levels using a Maxima First Strand cDNA Synthesis Kit (Cat#: K1672, Thermo Fisher Scientific) per the manufacturer’s instructions. QRT-PCR quantified gene expression in a master mix containing SYBR Green Supermix (Cat#: 1708884, Bio-Rad Inc., Hercules, CA, USA), primers (see Table 1), and 30 ng of cDNA per reaction under the following conditions: 3 min at 95 °C, followed by 40 cycles at 95 °C for 10 s and 60 °C for 40 s. GAPDH and 28S were used as internal loading controls.

PCR reactions were performed in a standard 96-well plate using the CFX96TM multicolor real-time PCR detection system (Bio-Rad Inc). For data analysis, the raw Ct value of each sample was first normalized to GAPDH or 28S to obtain dCt values. The dCt values were then calibrated to control cell samples to obtain ddCt. The fold change in expression was calculated using the standard 2 −^ddCt^ formula.

### 2.6. Extracellular Vesicle Isolation and Characterization

To assess IL13Rα2 levels in EVs, including large EVs and exosomes, tumor cells were cultured in serum-free medium for 72 h. Conditioned medium was collected and centrifuged at 300× *g* for 5 min, then at 2000× *g* for 20 min to remove cells and cellular debris. The supernatant was then centrifuged at 10,000× *g* for 1 h to pellet large EVs. The resulting supernatant was further ultracentrifuged at 100,000× *g* for 2 h to isolate exosomes. EV pellets were resuspended in 2× SDS sample buffer for Western blot analysis. For EV isolation used in CAR-T cell efficacy assays and for visualizing EV uptake by tumor cells, PBT neurospheres were cultured as described above for three days. Conditioned medium was collected and centrifuged at 300× *g* for 5 min, then at 2000× *g* for 20 min to remove cells and debris. The clarified supernatant was concentrated to approximately 0.5 mL using Vivaspin™ 20 centrifugal concentrators (Cat#: 28932363, Cytiva, Marlborough, MA, USA) with a 100 kDa molecular weight cutoff (MWCO), according to the manufacturer’s instructions. Concentrated samples were then loaded onto a qEV original 70 nm size-exclusion chromatography column (Cat#: ICO-70, Izon Science Ltd., Christchurch, New Zealand) for EV purification. Phosphate-buffered saline (PBS, Cat#: 9240, Irvine Scientific)-eluted EV fractions were further concentrated using Vivaspin™ 20 (100 kDa MWCO) to the desired final volume. The EV concentration was estimated by measuring total protein content using the Pierce™ BCA Protein Assay Kit (Cat#: 23225, Thermo Fisher Scientific). EV purification and morphology were verified by transmission electron microscopy (TEM, FEI Tecnai Twin 120 kV, Thermo Fisher Scientific, Hillsboro, OR, USA) at the City of Hope Electron Microscopy and Atomic Force Microscopy Core.

### 2.7. Flow Cytometry

Flow cytometry was used to assess the surface expression of IL13Rα2, CD63, and CD81 on GBM cells and EVs. For GBM cells, cells were washed with PBS containing 2% FBS and stained with anti-IL13Rα2 (Cat#: 354401, BioLegend, San Diego, CA, USA), CD63 (Cat#: NBP2-42225, Novus Biologicals), CD81 (Cat#: 349502, BioLegend) or isotype (Cat#: 400113, BioLegend) for 30 min at 4 °C. After incubation, cells were washed with PBS containing 2% FBS. Right before analysis on the MACSQuant (Miltenyi, Auburn, CA, USA), 4′,6-diamidino-2-phenylindole (DAPI, Cat#: D21490, Invitrogen) was added to assess viability. For isolated EVs, EVs were stained in PBS with anti-IL13Rα2 or isotype and incubated for 30 min at 4 °C. Samples were analyzed on the MACSQuant by first using PBS to verify the cleanliness of the MACSQuant. Isotype- and IL13Rα2-stained EVs were processed at low settings to avoid unnecessary background. For GBM cell analysis, each treatment was conducted in triplicate. Data was analyzed using either FlowJo (Tree Star, Version10.10.1) or FCS Express (De Novo Software, Version 7). Treatment under normoxia was used as a control.

### 2.8. Immunofluorescence Staining of Neurospheres

Six-day-cultured neurospheres were washed three times with PBS, fixed in 4% paraformaldehyde, and embedded in compound (Tissue-Tek, Torrance, CA, USA). Cryosections (10 µm) were prepared on a Leica CM1950 cryostat (Leica Biosystems, Wetzlar, Germany). After antigen retrieval with citrate buffer, sections were incubated with an anti-IL13Rα2 primary antibody (Cat#: AF146, R&D Systems), followed by the appropriate Alexa Fluor-conjugated secondary antibody. Slides were washed three times with PBS and mounted with antifade mounting medium containing DAPI for nuclear counterstaining. Fluorescent images were acquired on an EVOS^®^ FL Auto Imaging System (Thermo Fisher Scientific) using a 20× objective and identical acquisition settings across all experimental conditions to allow comparison of IL13Rα2 signal intensity.

### 2.9. Interaction of EV-Associated IL13Rα2 with GBM Cells

PBT030 cells were seeded onto four-well chamber slides (Cat#: 354104, BD Falcon, Bedford, MA, USA) and cultured overnight to reach approximately 40–50% confluence. Isolated EVs from PBT138/IL13Rα2-eGFP donor cells were added to each well at a final concentration of 16 µg/mL and gently mixed. After incubation for 4 h in cell culture incubator, cells were washed four times with EV-free PBS to remove unbound vesicles and cell debris. Fluorescence microscopy (Observer Z1, Carl Zeiss Microscopy, LLC, White Plains, NY, USA) with a 40× objective was used to acquire images and visualize the interaction of EV-associated IL13Rα2-eGFP with recipient GBM cells at the City of Hope microscopy core.

### 2.10. CAR T Cell Generation

IL13Rα2-specific CAR T cells were generated from healthy donor peripheral blood using the same CAR construct and procedures similar to those used clinically [33,34]. In brief, after density gradient centrifugation over Ficoll, the resulting PBMC underwent AutoMACS^®^ depletion to remove the CD25+ regulatory T cells and the CD14+ monocytes, followed by positive selection of the CD62L+ naïve memory (Tn/mem) population in accordance with the manufacturer’s instructions (Milteny Biotec, Auburn, CA, USA). Following enrichment, the Tn/mem cells were stimulated with Dynabeads Human T expander CD3/CD28 (Invitrogen) at a 1:3 T cell-to-bead ratio and transduced with IL13BBZ-T2A-CD19t lentivirus at an MOI of 0.5 in X Vivo15 containing 10% FCS with 5 μg/mL protamine sulfate (APP Pharmaceutical LLC, Schaumburg, IL, USA), 50U/mL rhIL-2 and 0.5 ng/mL rhIL-15. The resulting IL13Ra2-CAR T cells were expanded/maintained in culture medium supplemented with IL-2 and IL-15 three times per week.

### 2.11. CAR T Cell Cytotoxicity Assay

PBT138/IL13Rα2 cells were co-cultured with IL13Rα2-CAR T cells in 96-well plates at an effector-to-target (E:T) ratio of 1:5. Mock-transduced T cells served as the negative control. EVs from three sources were compared: (1) IL13Rα2-high exosomes derived from PBT138/IL13Rα2 cells, (2) IL13Rα2-intermediate derived from PBT030 with endogenous IL13Rα2 expression, and (3) IL13Rα2-low exosomes derived from PBT138/vector only-parental cells. To assess the impact of exosomal IL13Rα2 transfer, PBT138/IL13Rα2 cells were co-incubated with CAR T cells and EVs for three days throughout the assay. For the EV dose-dependent cytotoxicity assay, EVs from PBT138/IL13Rα2 cells were added at the start of co-culture at total protein concentrations of 1, 2, and 4 µg/mL. For the EV source-dependent cytotoxicity assay, we examined three EV types on PBT138/IL13Rα2 cells in the presence of IL13Rα2-CAR T cells. Treatments without EV application were used as controls. After three days of co-culture, luminescence was measured using a Spectra Max^®^ M3 multi-mode microplate reader (Molecular Devices, San Jose, CA, USA).

### 2.12. Ubiquitination Analysis

PBT030 and U-87 MG cells were seeded and cultured overnight to reach approximately 80% confluence before exposure to hypoxia (1% oxygen) or FBS deprivation (1% FBS). For the hypoxia treatment, tumor cells were first cultured under 1% oxygen or normoxic conditions for 24 h, and then MG132 (Cat#: S2619, Selleck Chemicals, Houston, TX, USA) was added at 0, 5, or 10 µM for an additional 6h incubation under normoxic or hypoxic conditions. For the 1% FBS deprivation treatment, tumor cells were cultured in 1% or 10% FBS medium for 48 h, and then 5 µM MG132 was added for an additional 10 h of incubation in 1% or 10% FBS medium. After treatment, cells were collected and analyzed by Western blotting to assess IL13Rα2 levels.

### 2.13. Statistical Analysis

All quantitative data are presented as the mean ± standard deviation (SD). Comparisons between two groups were performed using Student’s *t*-test, and comparisons among three or more groups were analyzed using one-way analysis of variance (ANOVA) with appropriate post hoc tests. Statistical analyses were conducted using GraphPad Prism version 8.0 (GraphPad Software). *p* values < 0.05 were considered statistically significant.

## 3. Results

### 3.1. Hypoxia Suppresses IL13Rα2 Expression in GBM Cells

**Figure 1:**
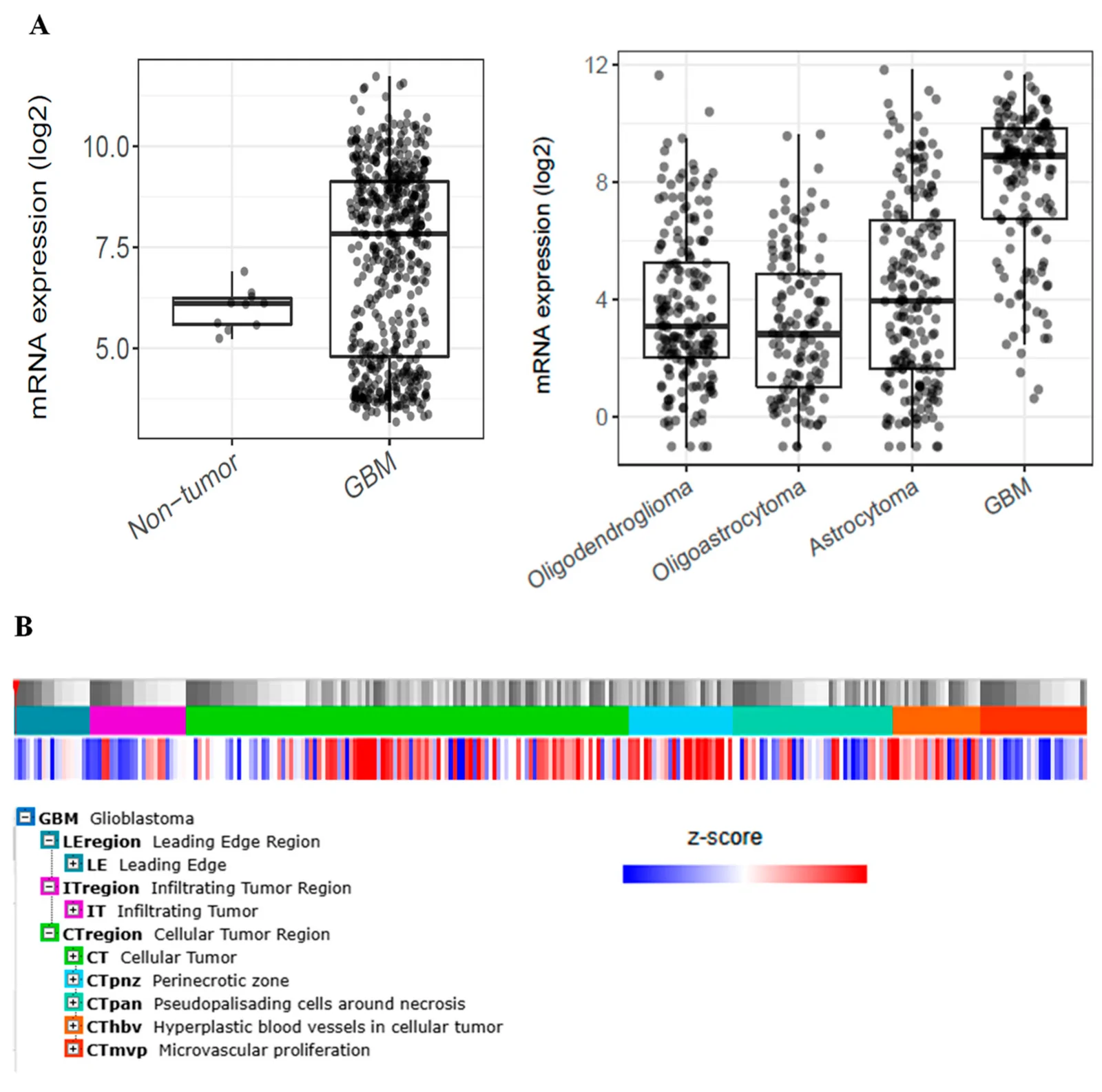
IL13Rα2 expression in human normal brain and tumor tissues. (**A**) IL13Rα2 expression is upregulated in glioma, as indicated by the GlioVis portal (https://gliovis.bioinfo.cnio.es/), accessed on 26 December 2025. (**B**) IL13Rα2 expression in different regions of GBM and its adjacent tissues was analyzed using the Ivy Glioblastoma Atlas Project (https://glioblastoma.alleninstitute.org/static/home, accessed on 26 December 2025).

IL13Rα2 is highly expressed in the majority of GBM tumors, a finding corroborated by our GBM cell lines and analyses of the GlioVis database and the Ivy Glioblastoma Atlas Project (Figure 1 and Figure S1). Notably, IL13Rα2 expression varies across tumor regions, with lower levels observed at the leading edge, in infiltrative tumor areas, and within pseudopalisading necrotic zones (Figure 1B), suggesting regulation by the heterogeneous tumor microenvironment. Given that hypoxia is a defining feature of the GBM microenvironment, we investigated its impact on IL13Rα2 expression.

**Figure 2:**
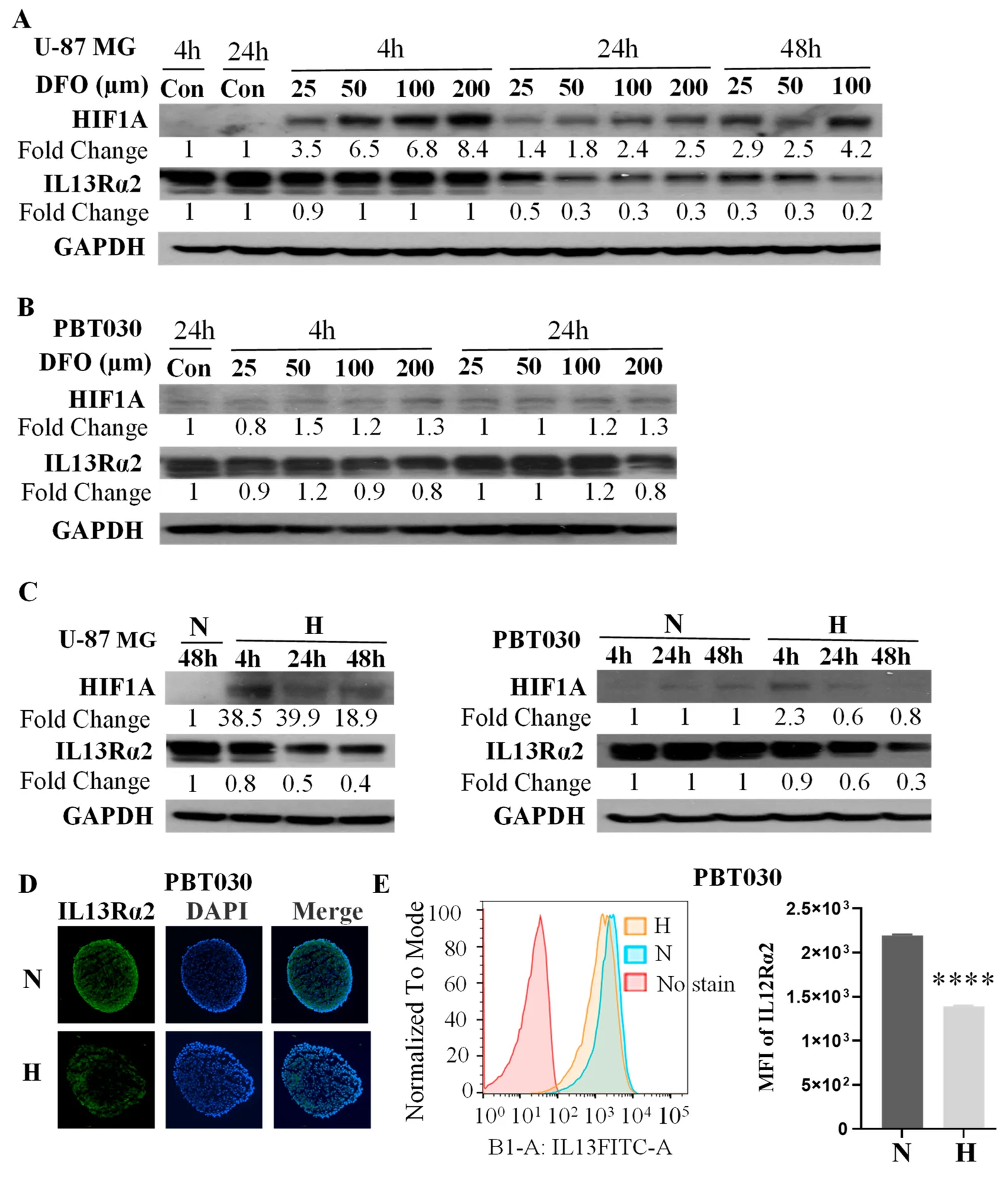
Hypoxia influences IL13Rα2 and HIF1A expression in GBM tumor cells. Western blotting of IL13Rα2 and HIF1A in U-87 MG (**A**) and PBT030 (**B**) cells treated with different concentrations of DFO at different time points, as well as under hypoxia (1% O_2_) (**C**), compared to the corresponding controls. IL13Rα2 on the PBT030 cell surface is decreased under hypoxic (1% O_2_) conditions for 48 h, as shown by immunostaining (**D**) and flow cytometry (flow histograms and median fluorescence intensity) (**E**). N: Normoxia; H: Hypoxia (1% oxygen). “****” Indicates *p* < 0.05. The original images of western blot are shown in Supplementary File S1.

We exposed GBM cells to hypoxic stress using the hypoxia-mimetic agent deferoxamine (DFO) or by culturing them under 1% O_2_, and we assessed IL13Rα2 expression by Western blotting, immunofluorescence, and flow cytometry (Figure 2). Under normoxic conditions, IL13Rα2 protein was readily detectable in both U-87 MG (Figure 2A) and the patient-derived brain tumor (PBT) cell line PBT030 (Figure 2B). DFO treatment produced a dose-dependent reduction in IL13Rα2 protein levels, with higher concentrations (e.g., 100 or 200 μM) over 24–48 h yielding a marked decrease relative to untreated controls. When U-87 MG and PBT030 cells were exposed to 1% O_2_ for 48 h, IL13Rα2 expression was substantially suppressed. Densitometric analysis of Western blots revealed that IL13Rα2 protein levels decreased to approximately 30% of normoxic levels in PBT030 cells and 40% in U-87 MG cells following 48 h of hypoxic exposure (Figure 2C).

To confirm the activation of hypoxic signaling, we evaluated HIF1α levels. As expected, DFO treatment stabilized HIF1α in a concentration-dependent manner, with robust induction observed at 100–200 μM. In contrast, under sustained hypoxia (1% O_2_), HIF1α levels peaked at early time points (4 h) and declined by 48 h, consistent with adaptive feedback mechanisms during chronic hypoxia. Despite this temporal pattern, IL13Rα2 downregulation remained pronounced, indicating that hypoxia suppresses IL13Rα2 expression independently of prolonged HIF1α stabilization.

Consistent with the total protein findings, surface IL13Rα2 expression was significantly reduced under hypoxic conditions. Immunofluorescence imaging revealed diminished membrane localization of IL13Rα2 in hypoxia-treated cells (Figure 2D). Flow cytometric analysis of PBT030 cells showed a ~34.3% decrease in the proportion of IL13Rα2-positive cells and an approximately 36.6% reduction in median fluorescence intensity (MFI) after 48 h at 1% O_2_ compared with normoxic controls (Figure 2E). Together, these data demonstrate that hypoxia suppresses IL13Rα2 expression at the protein level and reduces its surface availability.

At the transcript level, qRT-PCR analysis revealed decreased *IL13Rα2* mRNA expression in U-87 MG following 48 h of hypoxia (1% O_2_), using GAPDH and 28S as reference genes (Figure S2A). In contrast, PBT030 cells exhibited a less consistent transcriptional response (Figure S2B). In PBT138/IL13Rα2 cells, engineered to constitutively overexpress IL13Rα2 under the EF1 promoter (Figure S1), we also observed a decrease in IL13Rα2 mRNA levels after 48 h of hypoxia (Figure S2C), suggesting that low transcript abundance may be partly driven by post-transcriptional mechanisms. These findings differ from prior reports describing hypoxia-induced upregulation of *IL13Rα2* mRNA [22], suggesting that post-transcriptional regulation and microenvironment-specific factors contribute to the overall reduction in IL13Rα2 protein under prolonged hypoxic stress.

### 3.2. Nutrient and Growth Factor Deprivation Downregulates IL13Rα2 Expression

**Figure 3:**
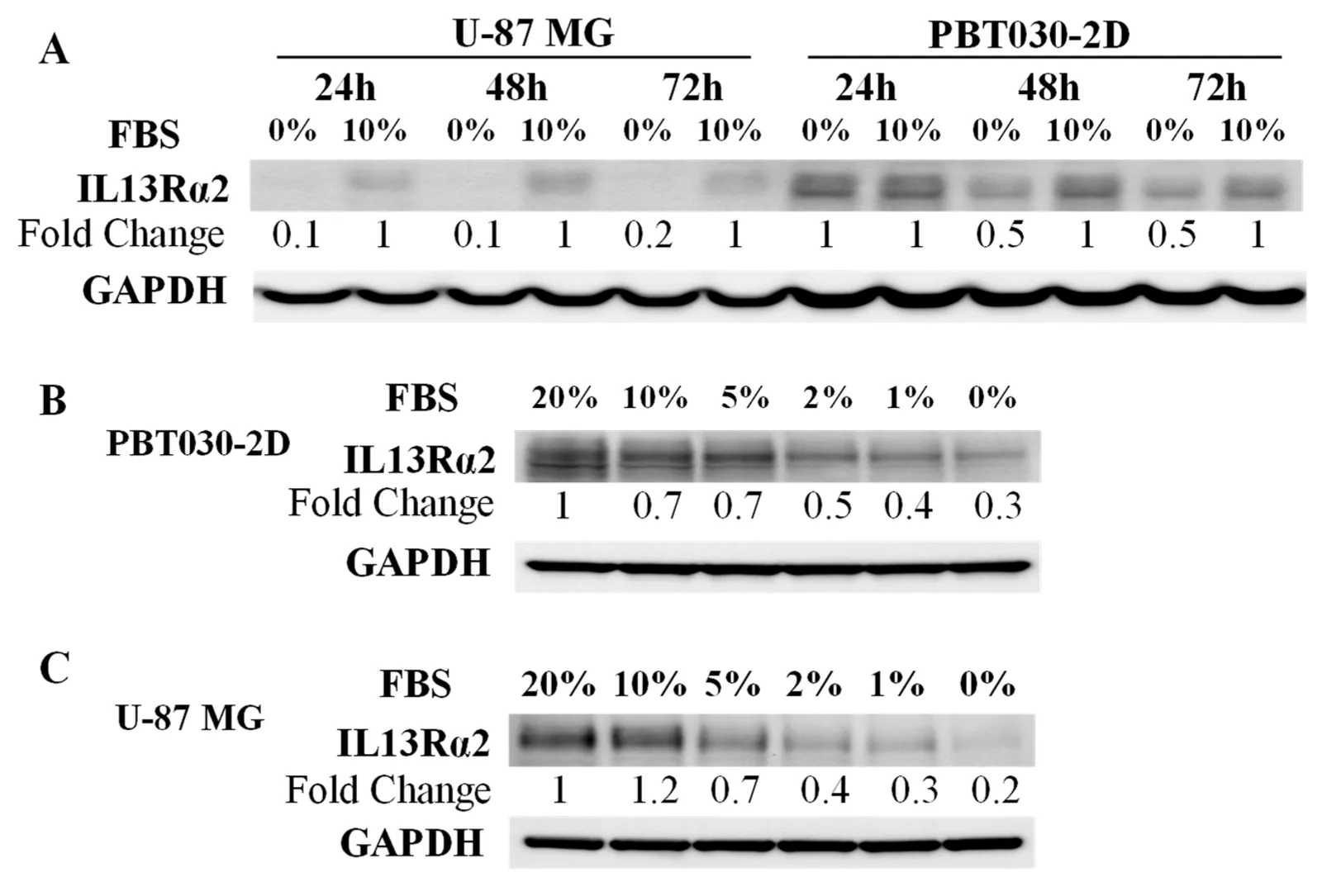
Reduced FBS results in decreased IL13Rα2 protein expression in GBM tumor cells. (**A**) IL13Rα2 expression in PBT030-2D (PBT030 monolayer culture) and U-87 MG cells cultured with medium containing 0% and 10% FBS for 24, 48, and 72 h via Western blotting. (**B**) IL13Rα2 expression in PBT030-2D cells cultured with different concentrations of FBS for 48 h. (**C**) IL13Rα2 expression in U-87 MG cells cultured with different concentrations of FBS for 48 h via Western blotting. GAPDH was used as a reference gene for loading control. 2D: Monolayer culture. The original images of western blot are shown in Supplementary File S1.

We next assessed whether nutrient and growth factor limitation, a hallmark of poorly vascularized tumor regions, influences IL13Rα2 expression in GBM cells. We cultured PBT030 and U-87 MG cells in media with decreasing FBS concentrations (20%, 10%, 5%, 2%, 1%, or 0%) for 24, 48, or 72 h, and we then evaluated IL13Rα2 protein levels by Western blotting. IL13Rα2 expression was highly sensitive to serum availability, with both cell lines demonstrating progressive reductions in protein levels under lower FBS conditions and with increasing duration, consistent with a time- and dose-dependent effect (Figure 3A–C).

In PBT030 cells, IL13Rα2 protein levels were reduced to approximately 29% of those observed in 10% FBS after 48 h in 2% FBS, and they became nearly undetectable under more stringent deprivation conditions (Figure 3B). U-87 MG cells, which exhibit lower baseline IL13Rα2 expression, showed a more modest reduction under the same conditions (Figure 3C). Intermediate decreases were observed at 5% FBS in both cell lines. GAPDH levels remained stable across all conditions, confirming equal protein loading and indicating that the observed changes were specific to IL13Rα2.

Consistent with these protein-level findings, qRT-PCR analysis showed that *IL13Rα2* mRNA expression in both PBT030-2D and U-87 MG cells decreased in a time- and dose-dependent manner under serum deprivation (Figure S3A–C). These data suggest that *IL13Rα2* expression in GBM cells depends on external growth factors and nutrients, and that deprivation induces downregulation, potentially through reduced proliferative signaling or the activation of cellular stress responses. This has important implications for tumor biology, as IL13Rα2 expression may be lowest in nutrient-deprived or necrotic regions, potentially limiting the efficacy of IL13Rα2-targeted therapies in these microenvironments.

Because both hypoxia and nutrient deprivation independently suppress IL13Rα2 expression, we next evaluated their combined effects. PBT030-2D and U-87 MG cells were cultured under varying FBS concentrations (10%, 5%, 2%, 1%, or 0%) and exposed to hypoxia (5% O_2_) for 48 h. Hypoxia alone reduced IL13Rα2 expression (Figure S4). When combined with serum deprivation, a synergistic reduction in IL13Rα2 protein levels was observed in PBT030-2D cells, whereas U-87 MG cells showed a less pronounced effect (Figure S4). These findings indicate that multiple microenvironmental stressors can cooperatively suppress IL13Rα2 expression, with cell line-specific variability in response.

### 3.3. IL13Rα2 Downregulation Is Reversible upon Reoxygenation and Nutrient Replenishment

**Figure 4:**
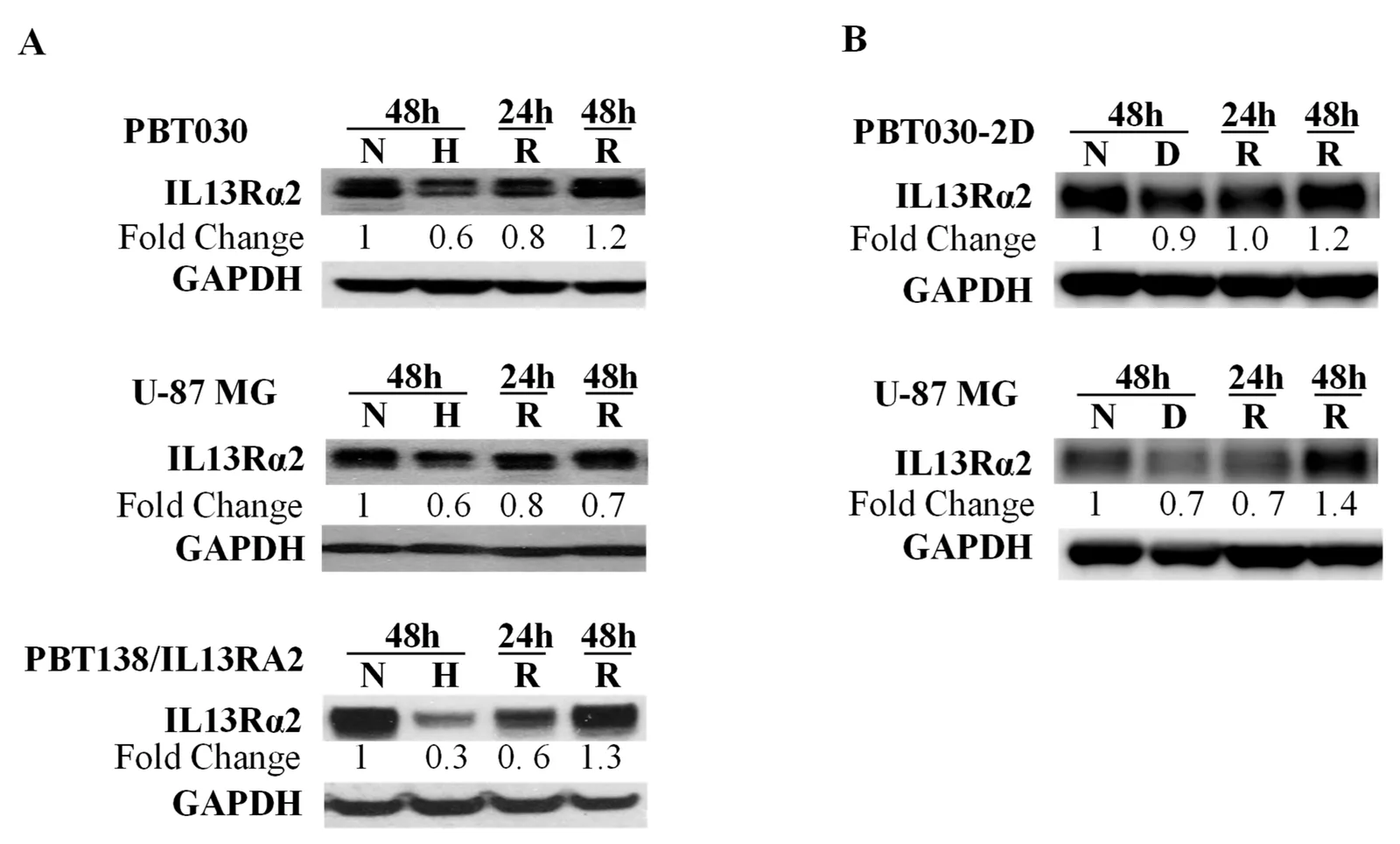
Restoration of IL13Rα2 expression in GBM cells under normal culture conditions post hypoxia or FBS deprivation treatments. (**A**) Western blot analysis of IL13Rα2 expression in PBT030, U-87 MG, and PBT138/IL13Rα2 (overexpression of IL13Rα2) cells exposed to hypoxia (1% O_2_) for 48 h, followed by reoxygenation under normoxic conditions for 24 or 48 h. (**B**) Western blot analysis of IL13Rα2 expression in PBT030 and U-87 MG cells cultured in culture medium containing 1% fetal bovine serum (FBS) for 48 h, followed by return to normal serum conditions for 24 or 48 h. GAPDH was used as a loading control. N: Normoxia; H: Hypoxia (1% oxygen); D: 1% FBS depletion; R: Restore to normal culture conditions; 2D: Monolayer culture. The original images of western blot are shown in Supplementary File S1.

We next investigated whether hypoxia- or nutrient deprivation-induced suppression of IL13Rα2 represents a permanent alteration or a reversible adaptive response. PBT030, U-87 MG, and PBT138/IL13Rα2 GBM cells were exposed to hypoxia (1% O_2_) for 48 h to suppress IL13Rα2 expression and were subsequently returned to normoxic conditions for defined recovery intervals (Figure 4A). Western blot analysis demonstrated progressive re-expression of IL13Rα2 following reoxygenation.

In PBT030 cells, IL13Rα2 protein expression began to recover within 24 h of returning to normoxia, reaching approximately 80% of the levels observed in continuously normoxic controls. By 48 h of reoxygenation, IL13Rα2 expression had returned to baseline levels and, in some experiments, modestly exceeded baseline expression (Figure 4A, upper panel). A comparable modest recovery pattern was observed in U-87 MG cells (Figure 4A, middle panel).

In PBT138/IL13Rα2 cells engineered to constitutively overexpress IL13Rα2, 48 h of hypoxia produced a marked reduction in both IL13Rα2 protein and mRNA expression, despite the presence of a strong ectopic promoter (Figure 4A, lower panel; Figure S2C). These findings suggest that hypoxia-mediated regulation of IL13Rα2 occurs, at least in part, through post-transcriptional and/or translational mechanisms. Following reoxygenation, IL13Rα2 expression progressively recovered over 24–48 h in these cells as well (Figure 4A, lower panel).

Collectively, these findings show that oxygen availability dynamically regulates IL13Rα2 expression and that hypoxia-induced low-IL13Rα2 states are reversible when normoxic conditions are restored. This reversible phenotype may be particularly relevant in GBM, where fluctuating oxygen gradients and cycling hypoxia are common features of the tumor microenvironment.

We next examined whether nutrient deprivation-induced suppression of IL13Rα2 expression was similarly reversible following nutrient replenishment. PBT030-2D and U-87 MG cells were cultured under serum-deprived conditions (1% FBS) to reduce IL13Rα2 expression and were then returned to standard growth media containing 10% FBS for defined recovery periods (Figure 4B). Western blot analysis demonstrated that low-serum-induced IL13Rα2 downregulation was reversible in both cell lines following the restoration of normal culture conditions. IL13Rα2 protein expression partially recovered within 24 h and returned to near-baseline expression by 48 h (Figure 4B), closely paralleling the recovery kinetics observed following reoxygenation after hypoxic exposure.

### 3.4. Ubiquitin-Mediated Degradation Potentially Drives IL13Rα2 Loss Under Hypoxic and Nutrient-Deprived Conditions

Our preceding results demonstrated that hypoxia and serum deprivation suppress IL13Rα2 protein expression. In addition to the observed reduction in *IL13Rα2* mRNA levels under these stress conditions, our prior RNA sequencing analysis of GBM cells cultured under hypoxia identified enrichment of genes involved in ubiquitin-mediated protein degradation pathways (Figure S6) [35]. These findings suggest that, beyond transcriptional regulation, post-translational mechanisms such as ubiquitination may also contribute to hypoxia-induced IL13Rα2 downregulation.

To directly test this hypothesis, PBT030 and U-87 MG cells were treated with the proteasome inhibitor MG132 under normoxic conditions or following exposure to hypoxia or FBS deprivation. Cells subjected to hypoxia or low-serum conditions were subsequently treated with MG132 for 6 h or 10 h, respectively.

**Figure 5:**
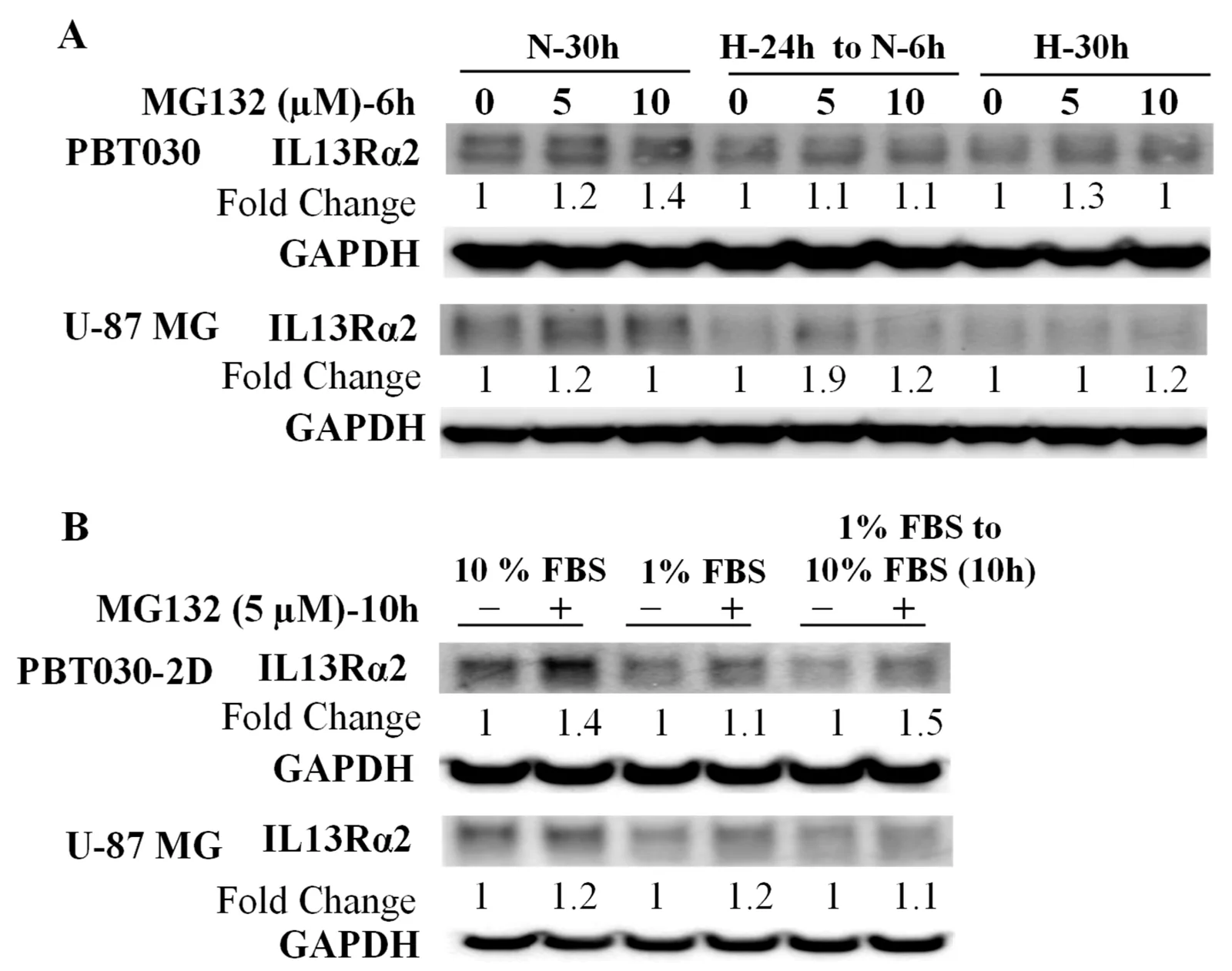
Ubiquitination is potentially involved in the downregulation of IL13Rα2 protein levels under stress conditions. (**A**) Western blot analysis of IL13Rα2 expression in PBT030 and U-87 MG cells treated with 1.0% hypoxia or normoxia for 24 h, then treated with MG132 at 5 or 10 µM for 6 h under normoxia or hypoxia. (**B**) Western blot analysis of IL13Rα2 expression in PBT030-2D and U-87 MG cells treated with culture medium containing 1% or 10% FBS for 48 h, then treated with 5 µM MG132 for 10 h in culture medium containing 1% or 10% FBS. GAPDH was used as a loading control. For each treatment comparison, samples treated with 0 μM MG132 (DMSO vehicle control) served as the corresponding control. N: Normoxia; H: Hypoxia (1% oxygen). The original images of western blot are shown in Supplementary File S1.

Under normal culture conditions, MG132 treatment for 6 h increased IL13Rα2 protein levels in both PB T030 and U-87 MG cells (Figure 5A). Importantly, MG132 partially restored IL13Rα2 expression in hypoxia-treated cells compared with untreated controls. These findings indicate that hypoxia-induced IL13Rα2 loss occurs potentially through proteasome-dependent degradation. A comparable pattern was observed under serum deprivation (Figure 5B), in which 10 h MG132 treatment suppressed IL13Rα2 degradation in both cell lines. Collectively, these results support a potential role for ubiquitin-mediated proteasomal degradation in the downregulation of IL13Rα2 under hypoxic and nutrient-deprived conditions.

### 3.5. Extracellular Vesicle-Mediated Trafficking of IL13Rα2 in GBM Cells

**Figure 6:**
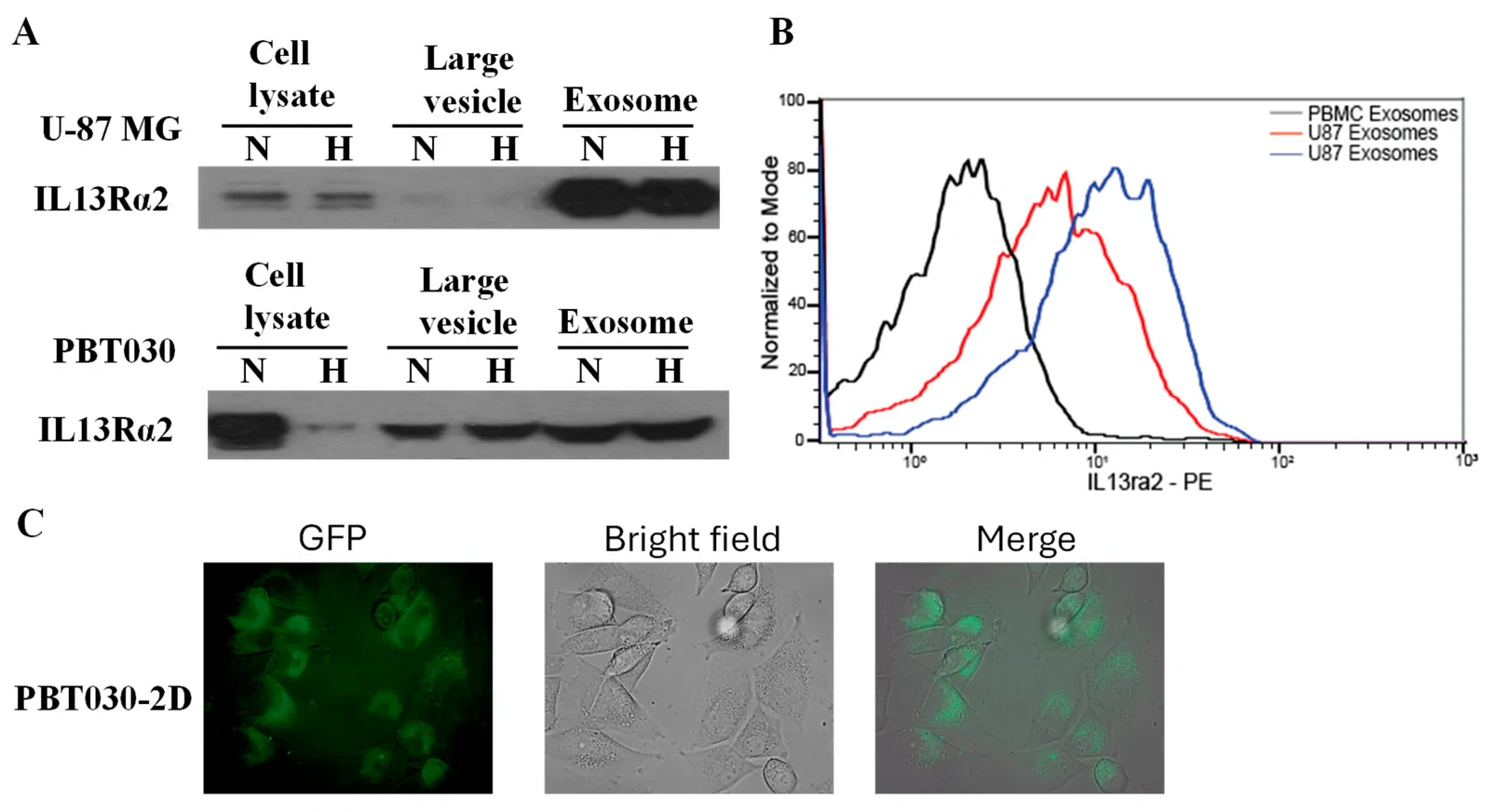
Extracellular vesicles containing IL13Rα2. (**A**) Western blot analysis of IL13Rα2 protein in cell lysates, large extracellular vesicles (10,000× *g* pellet), and exosomes (100,000× *g* pellet) isolated from U-87 MG and PBT030 cell culture media under normoxic (N) and hypoxic (H; 1% O_2_) conditions. Cell lysates were included as positive controls. (**B**) Flow cytometric analysis of IL13Rα2 expression on isolated U-87 MG-derived exosomes. Exosomes from PBMCs were used as a negative control (black line). Red and blue traces represent two independently isolated U-87 MG exosome preparations. (**C**) Representative fluorescence microscopy images demonstrating the interaction of extracellular vesicles (EVs) derived from PBT138 cells overexpressing human IL13Rα2-eGFP with recipient PBT030-2D cells following 4 h of co-incubation. GFP fluorescence indicates the presence of IL13Rα2+EVs within recipient cells. Corresponding bright-field and merged images are shown. The original images of western blot are shown in Supplementary File S1.

EVs are well recognized as efficient carriers of cellular cargo, including membrane proteins. We therefore investigated whether GBM cells release IL13Rα2 into the extracellular space via EVs. Using differential ultracentrifugation, we isolated large vesicles (10,000× *g* pellet) and small exosomal vesicles (100,000× *g* pellet) from conditioned medium of IL13Rα2-expressing GBM cells. Western blot analysis revealed the presence of IL13Rα2 in both large EV and exosome fractions (Figure 6A). In U-87 MG-derived samples, IL13Rα2 was readily detected in the exosome fraction and, to a lesser extent, in the large EV fraction. In contrast, PBT030 cells, which express higher baseline levels of IL13Rα2, showed robust IL13Rα2 signal in both vesicle populations.

These findings demonstrate that IL13Rα2, an integral membrane protein, can be incorporated into both plasma membrane-derived vesicles (microvesicles) and endosome-derived extracellular vesicles (exosomes). We next investigated whether oxygen tension influences IL13Rα2 vesicular release. Although total cellular IL13Rα2 expression decreased under hypoxic conditions, the relative abundance of IL13Rα2 in the exosome fraction appeared to increase. Specifically, PBT030 cells cultured under 1% O_2_ showed a slight increase in exosomal IL13Rα2 content, normalized to total exosomal protein, compared with normoxic controls (Figure 6A, lower panel), despite reduced overall cellular IL13Rα2 expression. These findings suggest that hypoxia may preferentially promote the packaging and/or release of IL13Rα2 into exosomes, potentially contributing to the depletion of IL13Rα2 from the tumor cell surface under hypoxic conditions.

We confirmed successful EV isolation by transmission electron microscopy and flow cytometry analysis of CD63 and CD81 expression (Figure S5A,B). Flow cytometric analysis of EVs isolated from U-87 MG cells further confirmed the presence of IL13Rα2 on EVs. Exosome-bead complexes stained positively for IL13Rα2 and showed a clear fluorescence shift relative to isotype control beads (Figure 6B), indicating the surface localization of IL13Rα2 while preserving extracellular domain accessibility.

To determine whether IL13Rα2-containing EVs interact with recipient tumor cells, we generated exosomes from PBT138 cells engineered to overexpress IL13Rα2 and eGFP as separate constructs. These IL13Rα2/eGFP-positive exosomes were incubated with recipient PBT030-2D cells, which express moderate endogenous IL13Rα2 levels. Fluorescence microscopy revealed GFP-positive puncta on the cell surface and within the intracellular compartment of recipient cells after 4 h of co-incubation (Figure 6C), consistent with EV binding and uptake. These findings provide proof of concept that IL13Rα2 can be transferred between GBM cells via EV-mediated mechanisms. Notably, PBT030 cells expressing endogenous IL13Rα2 also readily bound or internalized IL13Rα2/eGFP-positive exosomes, suggesting that EV-mediated transfer may also occur between IL13Rα2-positive tumor cells, potentially increasing overall IL13Rα2 antigen density at the tumor cell surface.

### 3.6. Exosomal Transfer of IL13Rα2 Enhances CAR T Cell-Mediated Tumor Killing

**Figure 7:**
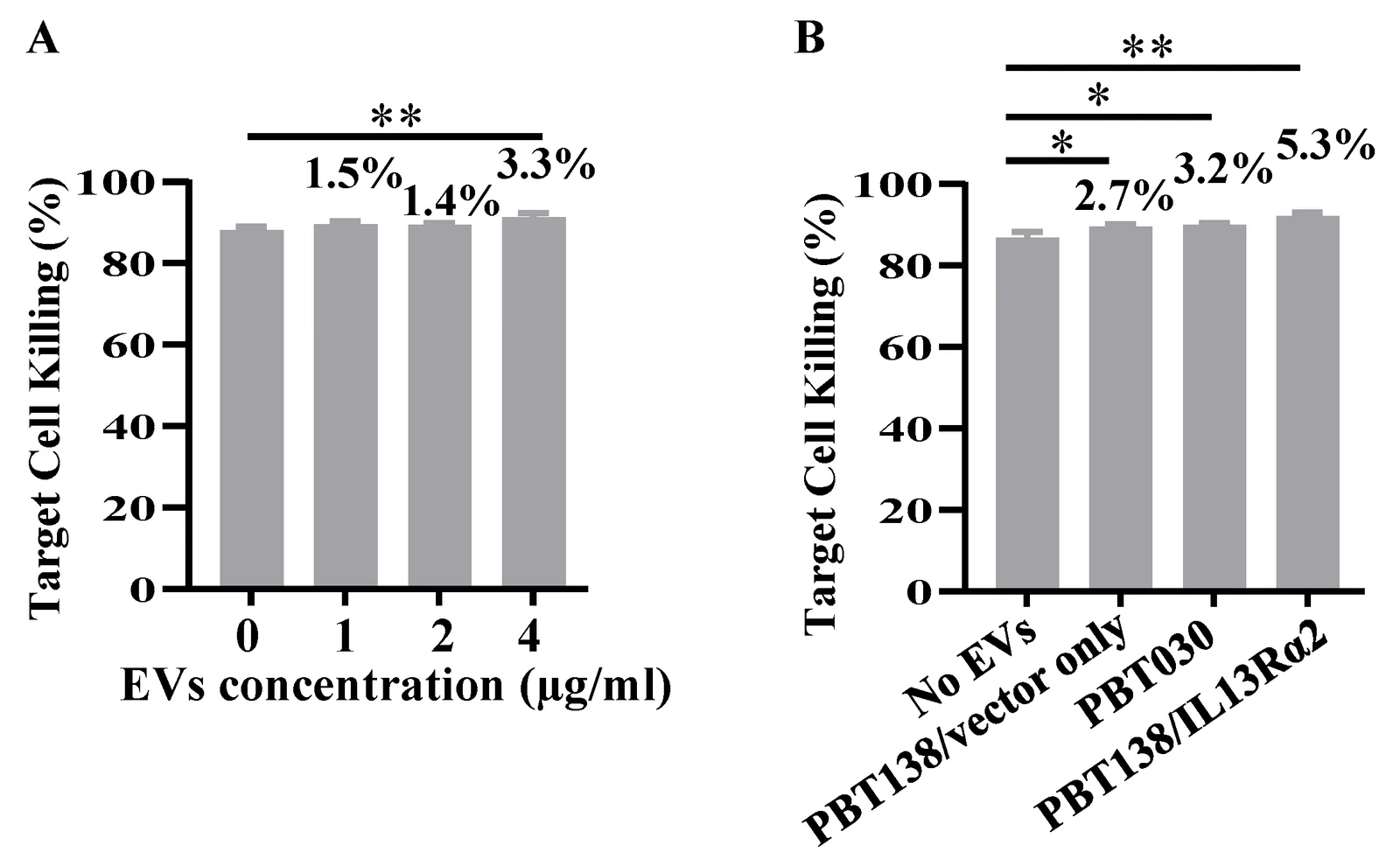
Increased IL13Rα2 via exosomes enhances CAR T cell killing efficacy against GBM cells. (**A**) CAR T cell-mediated cytotoxicity against PBT138/IL13Rα2 following treatment with increasing concentrations of EVs (0–4 μg/mL). Tumor cell viability was assessed by luminescence on day 3, and killing efficacy (%) was calculated by comparing each treatment group with its respective mock control. (**B**) CAR T cell-mediated cytotoxicity against PBT138/IL13Rα2 target cells following treatment with EVs (4 μg/mL) derived from different sources for 3 days. EV sources include: (1) exosomes from PBT138/IL13Rα2; (2) exosomes from PBT030 cells with endogenous intermediate IL13Rα2 expression; (3) exosomes from PBT 138/vector cells; and (4) no-exosome control. CAR T cells or mock-transduced T cells were co-cultured with target cells (20,000 cells per well) at an effector-to-target (E:T) ratio of 1:5. Data are presented as killing efficacy (%). Culture conditions used CAR T cell medium. Each treatment was conducted in quadruplicate. “*” and “**” Indicate *p* < 0.05.

We next investigated the functional significance of IL13Rα2 transfer via extracellular vesicles (EVs) in IL13Rα2-targeted CAR T cell immunotherapy. We initially hypothesized that IL13Rα2-positive exosomes might function as decoy antigens by binding IL13Rα2-specific CAR T cells and thereby reducing tumor cell killing. To test this, we performed cytotoxicity assays using IL13Rα2-CAR T cells and PBT138/IL13Rα2 GBM target cells. Target cells were co-cultured with IL13Rα2-CAR T cells at an effector-to-target (E:T) ratio of 1:5 in the presence or absence of IL13Rα2-containing EVs, and CAR T cell-mediated cytotoxicity was measured over time (Figure 7).

Contrary to our initial hypothesis, IL13Rα2-positive exosomes were able to modestly enhance CAR T cell-mediated tumor killing. In one representative experiment, adding different doses of exosomes (0, 1, 2, and 4 μg/mL) isolated from PBT138/IL13Rα2 (IL13Rα2-high exosomes) cells to co-cultures containing IL13Rα2-CAR T cells and PBT138/IL13Rα2 target cells significantly improved tumor cell killing over a three-day period in a dose-dependent manner (one-way analysis, *p* = 0.001). Specifically, even with an approximately 88% killing efficacy in the no-EVs control, adding IL13Rα2-high exosomes at 4 μg/mL increased CAR T cell cytotoxicity by approximately 3.3% compared with the treatment without exosomes (*p* = 0.03) (Figure 7A). Dose-escalation studies demonstrated that increasing concentrations of IL13Rα2-high exosomes progressively enhanced tumor cell killing.

We next examined whether EVs derived from cell lines expressing different levels of IL13Rα2 similarly influenced CAR T cell efficacy. These exosomes were isolated from IL13Rα2-expressing cells, including PBT138/IL13Rα2 cells (54.67% IL13Rα2-positive; IL13Rα2-high exosomes), endogenous IL13Rα2+ PBT030 cells (43.74% IL13Rα2-positive; IL13Rα2-intermediate exosomes), and PBT138/vector only cells (23.54% IL13Rα2-positive; IL13Rα2-low exosomes) (Figure S5C). As shown in Figure 7B, exosomes from IL13Rα2-low parental PBT138/vector-only cells slightly enhanced CAR T cell cytotoxicity by about 2.7% above baseline, indicating that the effect was not due to nonspecific immune stimulation by EVs alone but required IL13Rα2 cargo. Furthermore, IL13Rα2-high exosomes derived from two independent sources, PBT030 and PBT138/IL13Rα2 cells, demonstrated comparable enhancement of CAR T cell activity, with increases in killing efficacy of approximately 3.2% and 5.3%, respectively, further supporting IL13Rα2 as the critical functional determinant (one-way analysis, *p* < 0.0001). Additionally, IL13Rα2-positive exosomes did not exhibit toxicity toward CAR T cells or enhance cytotoxicity mediated by mock-transduced T cells, confirming that the observed effects depended on antigen-specific CAR engagement.

Collectively, these findings demonstrate that exosomal IL13Rα2 can functionally transfer antigenic material between tumor cells and enhance susceptibility to IL13Rα2-directed CAR T cell killing. This EV-mediated antigen transfer may broaden the population of CAR T cell-targetable tumor cells and supports a potential bystander killing mechanism in which IL13Rα2-positive tumor cells sensitize neighboring low-antigen or antigen-negative cells to CAR T cell therapy.

## 4. Discussion

Our study reveals two crucial and related facets of IL13Rα2 biology in GBM: (1) IL13Rα2 expression is dynamically regulated by microenvironmental conditions (hypoxia and nutrient restriction), and (2) IL13Rα2 protein can disseminate between tumor cells through extracellular vesicles, with potential implications for immunotherapy targeting this antigen. These findings extend our understanding of how GBM cells may evade or, conversely, be made more vulnerable to IL13Rα2-directed therapies under different tumor conditions.

We found that hypoxia and nutrient deprivation sharply downregulate IL13Rα2 in PBT and other GBM cell lines. Under chronic hypoxia (1% O_2_ for 48 h), IL13Rα2 protein levels decreased across multiple GBM cell models, including reduced surface presentation. Low serum (starvation of growth factors) similarly diminished IL13Rα2 expression. These results suggest that IL13Rα2 is a phenotype associated with well-oxygenated, nutrient-replete tumor cells, whereas cells in a stressed microenvironment (poorly perfused, hypoxic areas) may lose IL13Rα2. Given that hypoxia is a common feature of GBM pathology [36], this presents a potential mechanism of immune escape: IL13Rα2-targeted CAR T cells or drugs might be less effective in hypoxic tumor zones where the antigen is suppressed. Interestingly, our observations differ from some transcript-level studies, which reported induction of *IL13Rα2* mRNA in U-87 MG cells under acute hypoxia [22]. One possible explanation is that HIF1α or other hypoxia-responsive factors may initially induce transient upregulation during early hypoxic stress, whereas prolonged or chronic hypoxia subsequently reduces IL13Rα2 protein expression through combined transcriptional suppression, translational inhibition, and/or enhanced protein degradation. Hypoxia can trigger the unfolded protein response and ER stress pathways, which could affect IL13Rα2 folding or trafficking and potentially target it for degradation [37]. Our findings also indicated that ubiquitin-mediated proteasomal degradation potentially contributes to reduced IL13Rα2 expression under hypoxic and nutrient-deprived conditions. Further studies are needed to elucidate the underlying molecular mechanisms and to identify the E3 ubiquitin ligase(s) responsible for IL13Rα2 degradation. In addition, hypoxia reduced IL13Rα2 expression in PBT138/IL13Rα2 cells (which constitutively overexpress IL13Rα2) at both the protein and mRNA levels. These findings suggest that hypoxic stress may impact mRNA stability and/or that the promoter driving constitutive IL13Rα2 expression may not remain completely constitutive under hypoxic conditions. Further investigation will be required to elucidate the underlying mechanisms.

Additionally, our data suggest that EVs isolated from hypoxic conditions maintained or even enriched IL13Rα2 content within exosomes relative to normoxic conditions. Thus, even in settings where *IL13Rα2* mRNA expression is preserved or transiently increased, overall cell-associated IL13Rα2 protein may still decline because greater amounts of IL13Rα2 are being exported through EV shedding. These findings underscore the importance of evaluating protein-level regulation and vesicle-mediated trafficking, rather than relying on mRNA expression alone, when assessing tumor antigen availability for targeted therapies. These observations are consistent with prior findings by Lim et al., which demonstrated that hypoxic tumor microenvironments enhance extracellular vesicle production and alter EV cargo composition, thereby promoting adaptive tumor signaling and modulating the surrounding microenvironment [38].

Crucially, we demonstrated that the loss of IL13Rα2 under hypoxia is reversible. When hypoxic cells were reoxygenated, IL13Rα2 was re-expressed within days. This plasticity means that IL13Rα2-negative states induced by microenvironmental stress are not permanently fixed. In the GBM tumor context, regions of hypoxia can be spatially and temporally dynamic [39]. For instance, if a tumor area is revascularized or if therapy reduces cell density, previously hypoxic cells might regain IL13Rα2 expression. For IL13Rα2-CAR T therapy, this could have a silver lining: initial CAR T doses might primarily kill IL13Rα2-high cells, but by pruning those cells and possibly improving local oxygenation, they could allow formerly IL13Rα2-low cells to re-express the target, making them susceptible to subsequent CAR T cell infusions. A cyclical treatment strategy or a continuous presence of CAR T might exploit this dynamic. On the other hand, our findings also suggest that during treatment, if CAR T cells drive surviving tumor cells into a more stressed state (e.g., hypoxic), those cells may temporarily downregulate IL13Rα2 and evade killing as a form of reversible antigen escape. This is analogous to observations in other CAR T models where antigen loss or modulation contributes to relapse [29]. Our work adds microenvironment-driven antigen modulation as another layer to consider in antigen escape.

During tumor development and progression, solid tumors, particularly GBM, frequently generate hypoxic microenvironments due to rapid cellular proliferation and insufficient vascular supply [40]. Hypoxia, largely mediated through HIF-1 signaling pathways, induces broad metabolic reprogramming that alters cellular protein synthesis, lipid metabolism, and energy utilization [38,41]. These hypoxia-driven metabolic changes can substantially influence protein stability, post-translational processing, and membrane trafficking pathways, all of which may contribute to the regulation of IL13Rα2 expression. By incorporating patient-derived tumor sphere culture, we aimed to recapitulate this complex, dynamic tumor microenvironment and assess IL13Rα2 in a clinically relevant cellular context.

Our Western blot analyses demonstrated that complete serum deprivation (0% FBS) reduced IL13Rα2 expression more profoundly than 48 h of hypoxia exposure (5% oxygen) alone (Figure S4). This finding suggests that nutrient deprivation may suppress IL13Rα2 production more immediately than hypoxia itself. One possible explanation is that tumor cells exposed to hypoxia initially undergo adaptive metabolic reprogramming and continue to utilize residual nutrients in the culture medium before progressively reducing IL13Rα2 synthesis. In contrast, complete FBS deprivation directly eliminates essential growth factors, lipids, and metabolic substrates required for protein synthesis and cellular maintenance, resulting in a more rapid suppression of IL13Rα2 expression. These observations support the concept that both oxygen availability and nutrient status dynamically regulate IL13Rα2 expression through interconnected metabolic and stress-response pathways [42,43]. However, the underlying molecular mechanisms remain unclear. Future studies that incorporate transcriptional or translational inhibitors during reoxygenation or nutrient restoration may provide further insight into the reversibility of IL13Rα2 downregulation.

We also identified a novel mechanism through which EVs may transfer IL13Rα2 between tumor cells. Biochemical and flow cytometric approaches confirmed the presence of IL13Rα2 in exosomes and microvesicles from GBM cells. EV-mediated transfer of membrane proteins is increasingly recognized in cancer; for example, in triple-negative breast cancer, exosomes derived from mesothelin (MSLN)-targeted CAR T cells can impact the efficacy of MSLN-targeted therapies [44]. Our study of CAR T killing in GBM cells proposes that IL13Rα2-positive exosomes may transfer IL13Rα2 protein to other GBM cells, converting antigen-negative cells into antigen-positive cells (at least transiently at the cell surface). This finding has potential therapeutic ramifications. We demonstrated that such a transfer could enhance the effectiveness of CAR T cell killing. This is, to our knowledge, the first direct evidence that tumor-derived exosomes can facilitate a bystander killing effect for IL13Rα2 CAR T therapy by shuttling target antigen to antigen-negative tumor cells. The net result is an expanded range of tumor cell elimination despite heterogeneous antigen expression. Our observations are also supported by previous studies demonstrating that increasing antigen density on tumor cells enhances CAR T cell-mediated cytotoxicity, including γ-secretase inhibitor-mediated upregulation of BCMA in BCMA-targeted CAR T cell therapy and increased CD19 expression in CD19-targeted CAR T cell strategies [45,46]. These findings suggest that enhancing target antigen availability can improve CAR T cell efficacy across tumors with heterogeneous antigen expression. Nevertheless, despite these promising findings, our study has several limitations. Specifically, we employed a 2D culture system rather than a 3D culture model for the EV uptake and CAR T cell cytotoxicity assays to facilitate direct interactions between EVs and individual tumor cells and thereby maximize EV uptake efficiency. However, 2D and 3D culture conditions differ substantially and may influence cellular behavior, EV uptake, and CAR T cell function. Therefore, future studies should investigate how a more physiologically relevant 3D tumor microenvironment affects EV-mediated antigen modulation and CAR T cell activity.

Exosomes bearing IL13Rα2 could also have undesirable effects. In specific contexts, tumor-derived exosomes that bear target antigens or decoy receptors can act as sponges, sequestering therapeutic molecules. For instance, soluble or vesicle-bound BCMA can bind anti-BCMA CARs, reducing their availability to tumor cells, which is why preventing antigen shedding was beneficial [47]. In our system, one might worry that IL13Rα2^+^ exosomes could sequester IL13Rα2-CAR T cells or IL13-directed agents, thereby serving as decoys. However, our co-culture data did not show CAR T inhibition by IL13Rα2^+^ exosomes. Instead, the dominant outcome was modestly improved tumor-cell killing, although the increase in CAR T cell-mediated cytotoxicity after EV treatment was relatively small (approximately 3–5%), which may reflect the long treatment period (three days). Nevertheless, this effect still remains biologically relevant, as even modest increases in target antigen expression may enhance CAR T cell recognition and contribute to improved tumor cell elimination, particularly in tumors where antigen loss or low antigen density can limit therapeutic efficacy in a localized setting. This implies that exosomes could attach to nearby tumor cells (thereby delivering IL13Rα2 to them) rather than distracting CAR T cells. It is possible that in vivo, exosomes could diffuse and bind CAR T cells or other sinks; the balance between these effects would determine the net impact. Notably, a report indicated that blocking tumor EV secretion can improve CAR T cell function [48], presumably by preventing EV-mediated immunosuppression or decoy activity. In the case of IL13Rα2, because humans lack a proper soluble form [49], exosomal shedding is the primary route of antigen release, which can be either advantageous (spreading the antigen) or detrimental (reducing cell-surface antigen or diverting CAR T cells). Further investigation is warranted to examine the effect of short-period co-culture treatment of EVs on CAR T cells killing efficacy, and to understand the abundance of IL13Rα2-bearing exosomes in GBM patients and their in vivo interaction with infused CAR T cells.

Our findings also have implications for diagnostics and tumor monitoring. The detection of IL13Rα2-positive exosomes in patient plasma has been proposed as a liquid biopsy marker for GBM [45]. Our results validate that tumor cells indeed secrete IL13Rα2 into EVs. It would be interesting to see whether IL13Rα2 levels in circulating exosomes correlate with tumor burden or with IL13Rα2 expression in the tumor, as suggested by a study of peripheral nerve tumors [27]. Moreover, if hypoxia increases IL13Rα2 exosome release, as our data hint, then circulating IL13Rα2 might also reflect hypoxic tumor activity. Clinically, one might monitor IL13Rα2 EV levels during IL13Rα2-targeted therapy; a drop in IL13Rα2-positive EVs might indicate antigen loss in the tumor (and thus emerging resistance), whereas stable or rising EV antigen could indicate continued target expression.

## 5. Conclusions

In summary, this study highlights a nuanced interplay between the tumor microenvironment and the expression of a key GBM antigen (IL13Rα2) in both patient-derived tumor models and commercially available GBM cell lines. Hypoxia and low nutrients suppress IL13Rα2, potentially creating transient CAR T resistance in those regions, but reoxygenation and nutrient replenishment can restore antigen expression. Simultaneously, tumor-derived exosomes carrying IL13Rα2 can bridge antigen-positive and antigen-negative cells, enabling CAR T cells to achieve more complete tumor cell killing than would be expected from an antigen profile. These insights suggest several actionable strategies for clinical implementation: combining IL13Rα2-CAR T therapy with treatments that alleviate hypoxia or with epigenetic/transcriptional modulators to boost IL13Rα2 expression could prevent antigen-low escape. Alternatively, leveraging the vesicle pathway, for example, by supplementing patients with IL13Rα2-loaded exosomes or nanoparticles, could mark otherwise negative tumor cells for destruction by CAR T cells. This concept parallels the oncolytic virus marking strategy [46] but uses a cell-derived delivery system. Any such approach would need to ensure specificity and safety; however, given the low basal expression of IL13Rα2 [50], off-tumor effects might be limited. Finally, our work underscores the importance of considering tumor physiological conditions, such as hypoxia and nutrient status, in both the design and timing of immunotherapies. Therapies that can adapt to or exploit the tumor’s dynamic antigen landscape will likely be more successful in achieving durable GBM remissions.

## Figures and Tables

**Table 1:** Primer list of qRT-PCR.

GAPDH RT-F	5′ AGGTGAAGGTCGGAGTCAAC 3′
GAPDH RT-R	5′ ATCTCGCTCCTGGAAGATGG 3′
28S RT-F	5′ GTCGGCTCTTCCTATCATTGTG 3′
28S RT-R	5′ CGCAACAACACATCATCAGTAGG 3′
IL13Rα2 RT-F	5′ GTGGAGTGATAAACAATGCTGGG 3′
IL13Rα2 RT-R	5′ TGGGTAGGTGTTTGGCTTACGC 3′
Inserted IL13Rα2 RT-F	5′ AGGTGCAGGACATGGACTGCGTGTA 3′
Inserted IL13Rα2 RT-R	5′ TAGAACAGGTTGTAGTTGGTGTC 3′

## Data Availability

The original contributions presented in this study are included in the article. Further inquiries can be directed to the corresponding author.

## Supplementary Materials

The following supporting information can be downloaded at: https://www.mdpi.com/article/10.3390/cancers18213513/s1, Figure S1. IL13Rα2 expression in Different Human Brain Normal and Tumor Cell Lines. Western blotting assay of IL13Rα2 expression in different GBM cell lines and astrocyte, 2D: Monolayer culture; 3D: Sphere culture. GAPDH was used as a loading control. Figure S2. Hypoxia suppresses *IL13Rα2* mRNA expression in GBM tumor cells. QRT-PCR analysis for *IL13Rα2* expression in U-87 MG (A), PBT030 (B), and PBT138/IL13Rα2 (C) cells treated with 1% O_2_ for 48 h. GAPDH and 28S were used as reference genes for normalization. Each treatment was conducted in triplicate. 2D: Monolayer culture; 3D: Sphere culture. Figure S3. FBS deprivation suppresses *IL13Rα2* mRNA expression in GBM tumor cells. (A) qRT-PCR analysis of *IL13Rα2* expression in U-87 MG cells cultured in DMEM supplemented with 0% or 10% FBS for 24, 48, and 72 h. (B) qRT-PCR was performed to assess *IL13Rα2* expression in U-87 MG cells cultured in DMEM supplemented with serial concentrations of FBS for 48 h. (C) qRT-PCR assay was performed to assess *IL13Rα2* expression in PBT030-2D cells cultured in DMEM supplemented with different concentrations of FBS for 48 h. GAPDH was used as a reference gene for normalization. Each treatment was conducted in triplicate. 2D: Monolayer culture. Figure S4. IL13Rα2 expression in GBM cells after FBS deprivation alone or in combination with 5% O_2_ hypoxia. Western blot assay of IL13Rα2 expression in PBT030-2D and U-87 MG cells treated with different concentrations of FBS alone or in combination with 5% O_2_ for 48 h. GAPDH was used as a loading control. 2D: Monolayer culture. Figure S5. Qualification and verification of IL13Rα2 on the surface of EVs. (A) Representative electron microscopy (EM) image of EVs (A) and flow cytometry analysis of CD63 and CD81 expression (B) on the EVs, confirming successful EV isolation and validating the EV isolation method. (C) Flow cytometry analysis detecting IL13Rα2 on the surface of EVs purified from PBT138/vector only, PBT030, and PBT138/IL13Rα2 cell lines. Figure S6. Ubiquitin-mediated proteolysis pathway generated from RNA sequencing data in PBT030 cells treated under hypoxic (1% O_2_) conditions for 48 h. Genes highlighted in the red box are upregulated under hypoxic conditions; those in the green box are downregulated; and those in the gray box are not significantly affected.

## Abbreviations

The following abbreviations are used in this manuscript:

IL13Rα2	Interleukin-13 Receptor alpha 2
GBM	Glioblastomas
CAR T	Chimeric Antigen Receptor T
EVs	Extracellular Vehicles
DFO	Desferrioxamine
FBS	Fetal Bovine Serum
PBT	Patient-derived Brain Tumor
ATCC	American Type Culture Collection
EGF	Epidermal Growth Factor
FGF	Fibroblast Growth Factor
MWCO	Molecular Weight Cutoff
DAPI	4′,6-Diamidino-2-phenylindole
OCT	Optimal Cutting Temperature
TEM	Transmission Electron Microscopy
SD	Standard Deviation
ANOVA	Analysis of Variance
CSF	Cerebrospinal Fluid
MFI	Median Fluorescence Intensity
