# Supplementary material for: Microenvironmental IL13 Rα2 Expression and Enable Extracellular Vesicle-Mediated Antigen Transfer in Glioblastoma
Source: Cancers (Basel). 2026 Oct 31;18(21):3513. doi: 10.3390/cancers18213513 (PMC13761624; doi:10.3390/cancers18213513)
Supplement: Supplementary file 1 [file cancers-18-03513-s001.zip › cancers-4545525-supplementary.pdf]

Figure S1. IL13Rα2 expression in Different Human Brain Normal and Tumor Cell Lines.

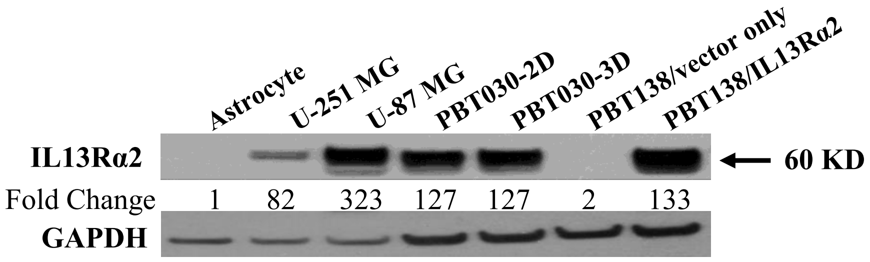

Figure S1. IL13Rα2 expression in Different Human Brain Normal and Tumor Cell Lines. Western blotting assay of IL13Rα2 expression in different GBM cell lines and astrocyte, 2D: Monolayer culture; 3D: Sphere culture. GAPDH was used as a loading control. .

Figure S2. Hypoxia Suppresses *IL13Rα2* Expression in GBM Tumor Cells at mRNA Level

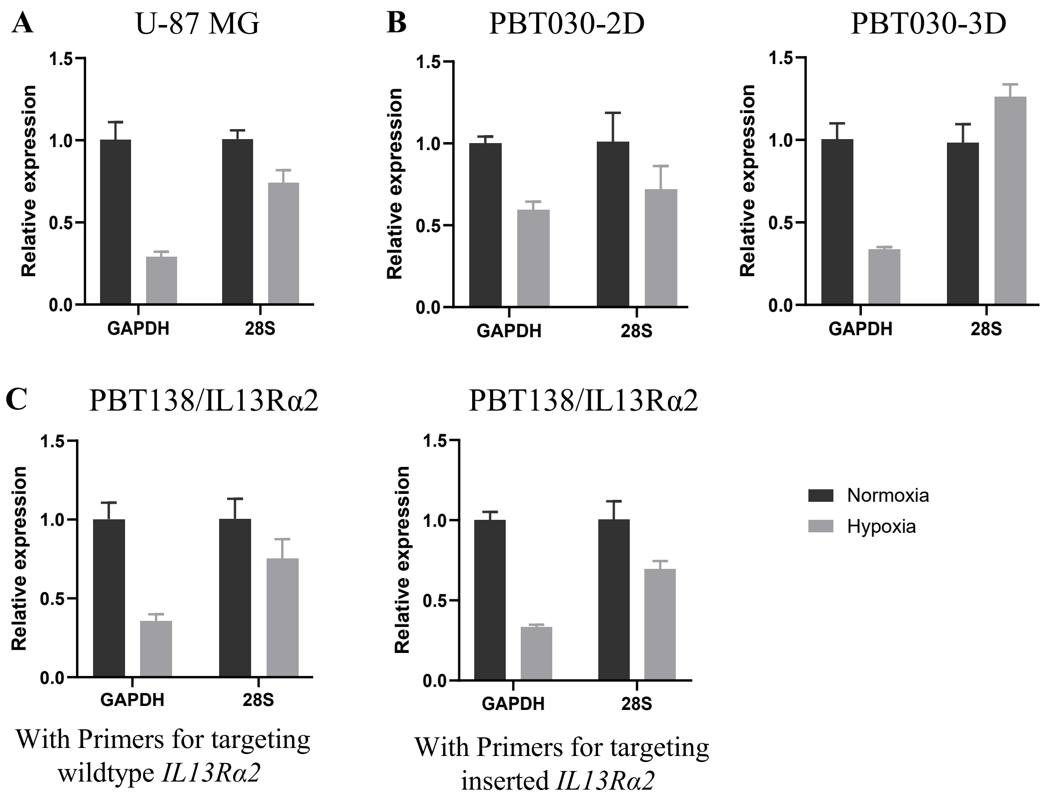

Figure S2. Hypoxia suppresses *IL13Rα2* mRNA expression in GBM tumor cells. QRT-PCR analysis for *IL13Rα2* expression in U-87 MG (A), PBT030 (B), and PBT138/IL13Rα2 (C) cells treated with 1% O_2 for 48 h. GAPDH and 28S were used as reference genes for normalization. Each treatment was conducted in triplicate. 2D: Monolayer culture; 3D: Sphere culture.

Figure S3. FBS deprivation suppresses *IL13Rα2* mRNA expression in GBM tumor cells.

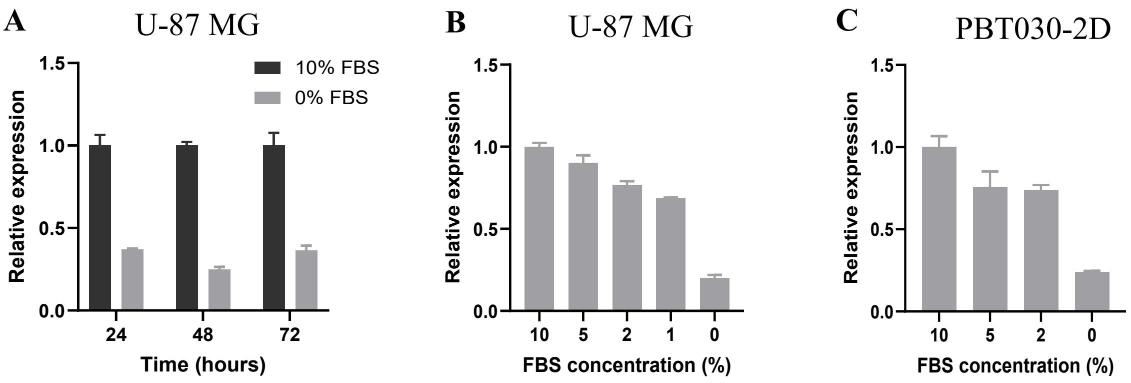

Figure S3. FBS deprivation suppresses *IL13Rα2* mRNA expression in GBM tumor cells. (A) qRT-PCR analysis of *IL13Rα2* expression in U-87 MG cells cultured in DMEM supplemented with 0% or 10% FBS for 24, 48, and 72 h. (B) qRT-PCR was performed to assess *IL13Rα2* expression in U-87 MG cells cultured in DMEM supplemented with serial concentrations of FBS for 48 h. (C) qRT-PCR assay was performed to assess *IL13Rα2* expression in PBT030-2D cells cultured in DMEM supplemented with different concentrations of FBS for 48 h. GAPDH was used as a reference gene for normalization. Each treatment was conducted in triplicate. 2D: Monolayer culture.

Figure S4. IL13Rα2 expression in GBM cells after FBS deprivation alone or in combination with 5% O_2 hypoxia.

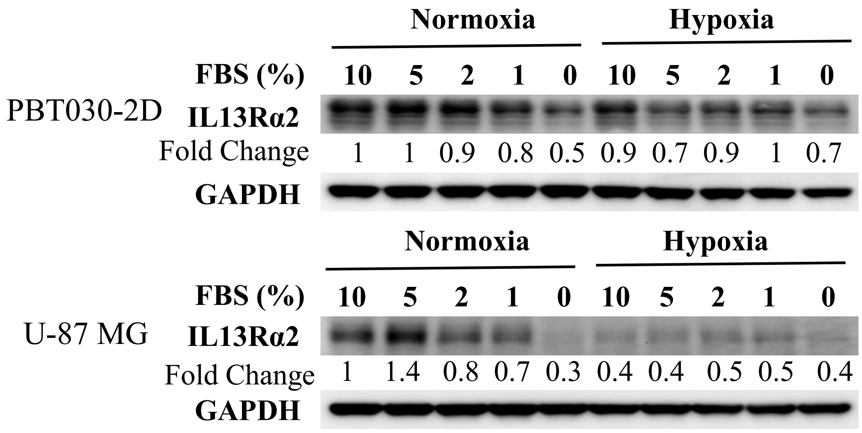

Figure S4. IL13Rα2 expression in GBM cells after FBS deprivation alone or in combination with 5% O_2 hypoxia. Western blot assay of IL13Rα2 expression in PBT030-2D and U-87 MG cells treated with different concentrations of FBS alone or in combination with 5% O_2 for 48 h. GAPDH was used as a loading control. 2D: Monolayer culture.

Figure S5. Qualification and Verification of IL13Rα2 on the Surface of EVs

A

PBT030

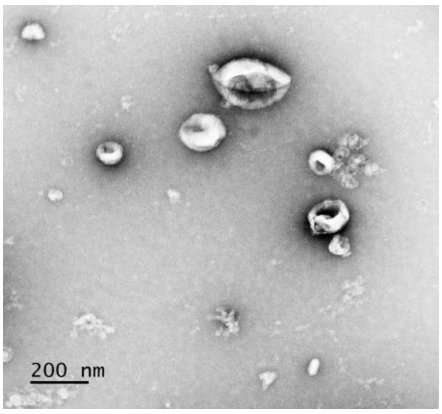

B

PBT030 | PBT138/vector only | PBT138/IL13Rα2

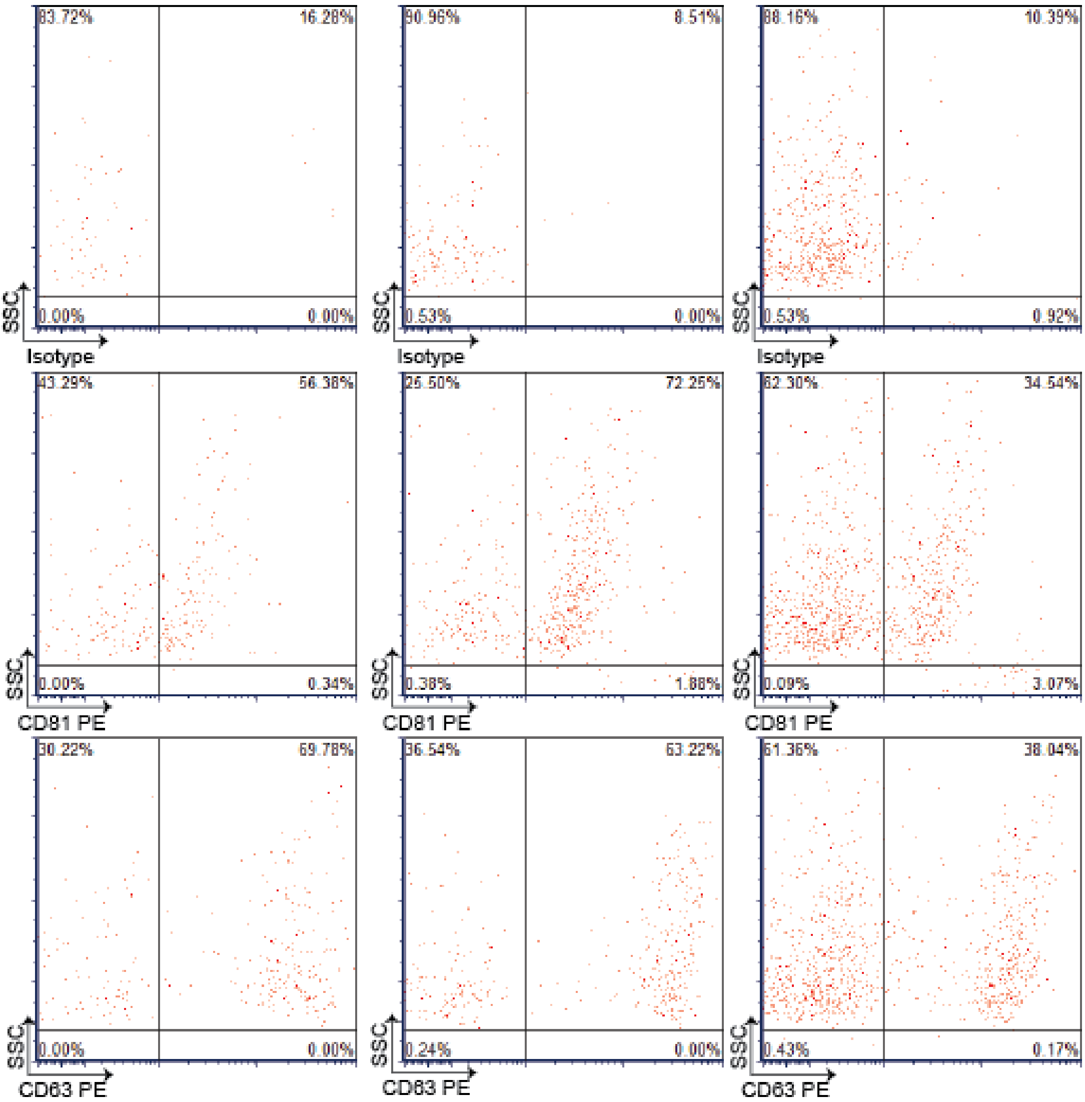

Figure S5. Qualification and Verification of IL13Rα2 on the Surface of EVs

C

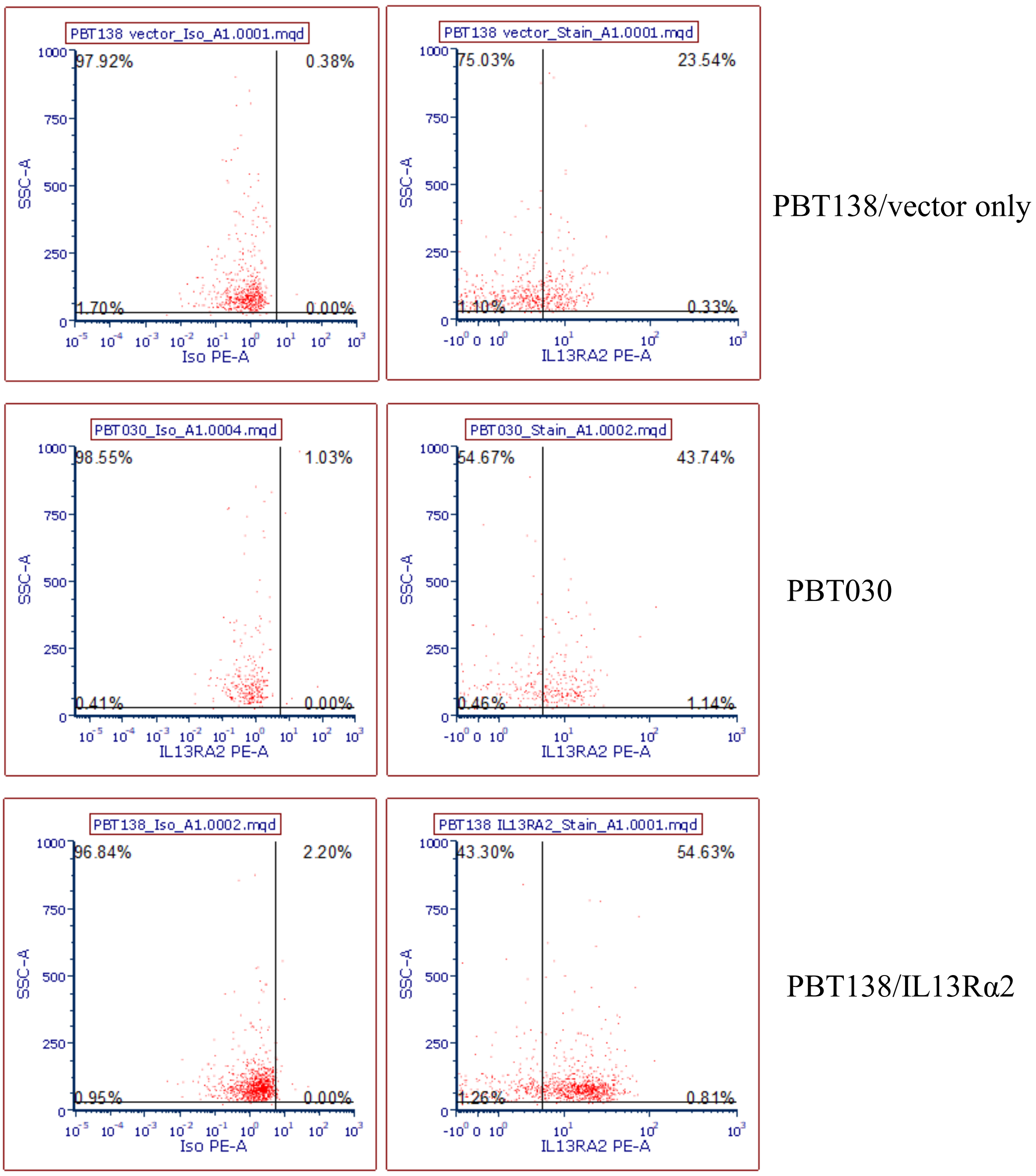

Figure S5. Qualification and verification of IL13Rα2 on the surface of EVs. (A) Representative electron microscopy (EM) image of EVs (A) and flow cytometry analysis of CD63 and CD81 expression (B) on the EVs, confirming successful EV isolation and validating the EV isolation method. (C) Flow cytometry analysis detecting IL13Rα2 on the surface of EVs purified from PBT138/vector only, PBT030, and PBT138/IL13Rα2 cell lines.

Figure S6. Ubiquitin-mediated proteolysis pathway generated from RNA sequencing data in PBT030 cells treated under hypoxic (1% O_2) conditions.

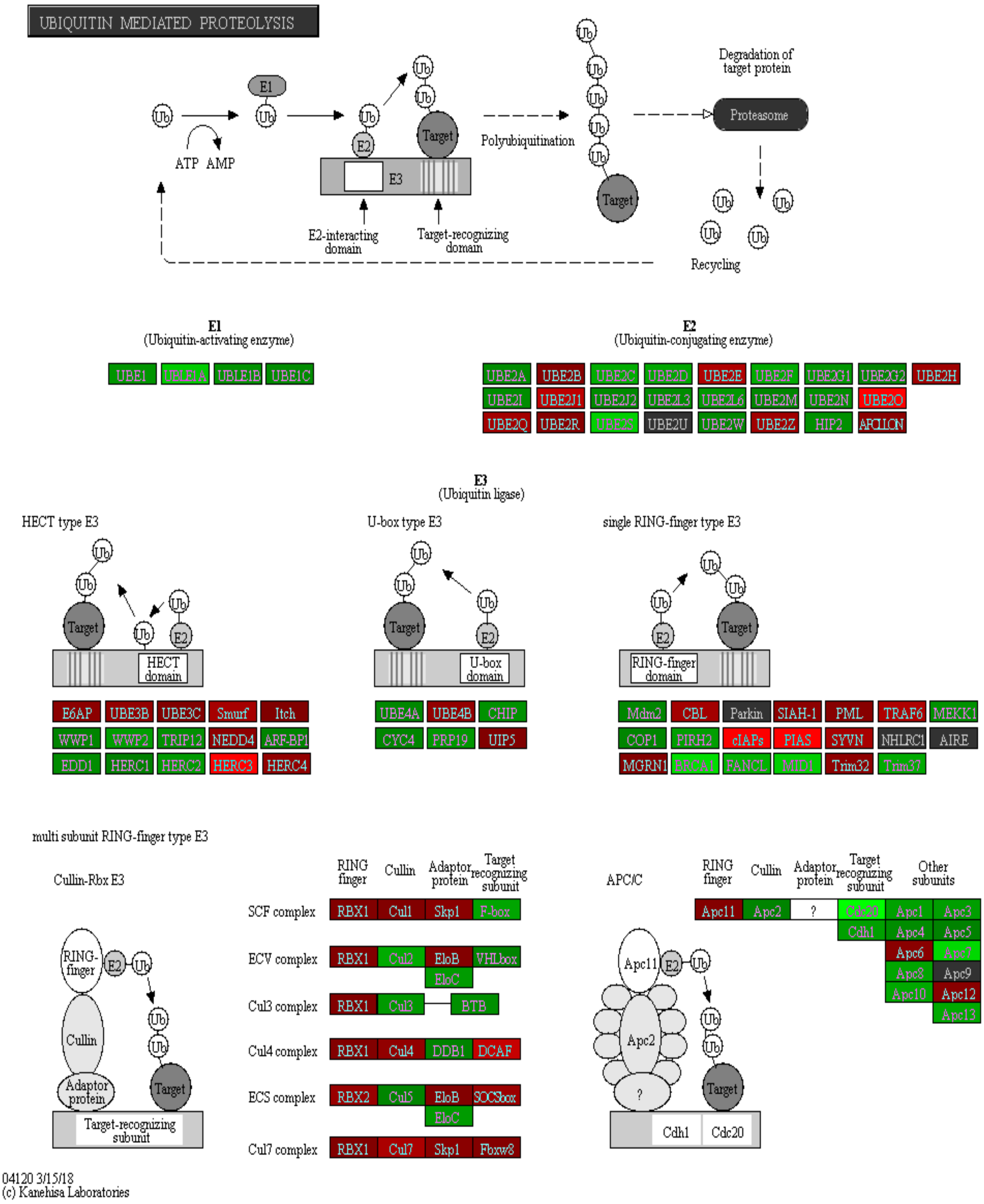

Figure S6. Ubiquitin-mediated proteolysis pathway generated from RNA sequencing data in PBT030 cells treated under hypoxic (1% O_2) conditions for 48 h. Genes highlighted in the red box are upregulated under hypoxic conditions; those in the green box are downregulated; and those in the gray box are not significantly affected.
